# Transcriptomic responses of beet to infection by beet mild yellowing virus

**DOI:** 10.1186/s12870-025-07514-6

**Published:** 2025-10-21

**Authors:** Vinitha Puthanveed, Radha Sivarajan Sajeevan, Abu Bakar Siddique, Erik Alexandersson, Pratikshya Joshi, Per Snell, Britt-Louise Lennefors, Anders Kvarnheden

**Affiliations:** 1 Department of Plant Biology, Swedish University of Agricultural Sciences, Box 7080, Uppsala, 750 07 Sweden; 2 Department of Plant Protection Biology, Swedish University of Agricultural Sciences, Box 190, Lomma, 234 22 Sweden; 3 Department of Plant Breeding, Swedish University of Agricultural Sciences, Box 190, Lomma, 234 22 Sweden; 4 DLF Beet Seed AB, Säbyholmsvägen 24, Landskrona, 261 91 Sweden; 5 United Beet Seeds, Industriepark 15, Tienen, 3300 Belgium

**Keywords:** Differential gene expression, Endoplasmic reticulum mediated stress tolerance, Transcriptome response, Virus resistance, Virus yellows

## Abstract

**Background:**

Virus yellows (VY) disease of sugar beet is caused by a complex of aphid-transmitted viruses, including beet mild yellowing virus (BMYV). Neonicotinoids have been used for preventing VY through aphid management, but with the recent ban on neonicotinoids in Europe, the risks for outbreaks of VY have increased dramatically. To study the host responses to BMYV infection and identify the differentially expressed genes (DEGs), we conducted an RNAseq experiment using a resistant genotype of wild beet and a susceptible breeding line of sugar beet. The experiment contained four plant treatments: exposure to aphids with or without BMYV, only insecticide spray and untreated control. Leaves were collected for analyses at 0, 1, 4, 14, 21 and 28 days post-inoculation (DPI).

**Results:**

Following BMYV inoculation, resistant plants did not show any chlorosis even at 28 DPI, whereas susceptible plants displayed typical virus symptoms. Using RT-qPCR, BMYV was detected already at 1 DPI in both genotypes. At 14, 21 and 28 DPI, the virus titre in young and inoculated leaves of the susceptible genotype was higher. RNAseq revealed more DEGs as a response to BMYV infection for the susceptible genotype. In inoculated leaves, the number of DEGs increased faster for the susceptible genotype, while in young leaves, the trend was similar for susceptible and resistant genotypes. This shows that the plant responses in inoculated leaves to virus infection appeared at a larger scale in the susceptible genotype. Seven of the significantly upregulated genes in the resistant genotype encoded proteins involved in protein processing in the ER. This could be one mechanism contributing to the absence of symptoms in this genotype.

**Conclusions:**

This study offers new insights into the transcriptomic events and genetic pathways regulating the defence response to BMYV in a partially resistant genotype. We present 14 candidate genes for partial resistance to BMYV and one of the possible mechanisms contributing to reduced virus levels and absence of symptoms. The findings will be of importance for future functional studies to understand the mechanisms of resistance and susceptibility as well as for the breeding of BMYV resistance.

**Supplementary Information:**

The online version contains supplementary material available at 10.1186/s12870-025-07514-6.

## Background

Sugar beet (*Beta vulgaris* ssp. *vulgaris*) is a tuber crop of the family Amaranthaceae that is cultivated mainly in the temperate regions of the world [1, 2]. It is a diploid species with 18 chromosomes (2n = 18) and a genome size of 714–758 mega base pairs [3, 4]. Sugar beet has become one of the major crops for sugar production besides sugar cane, and it accounts for 20% of the sugar production in the world [5].

High yielding varieties is a priority for sugar beet breeding programs across the world. However, in order to attain the full potential of the crop, several secondary traits like bolting resistance, germination potential and resistance to pests and diseases are equally important [6]. Sugar beet breeding in the public and private sectors focuses mainly on resistance traits [7–10]. Through breeding programs, important traits have been successfully introgressed to sugar beet from wild species, especially disease resistance [6, 11], e.g., resistance against beet cyst nematode (*Heterodera schachtii*) from *Beta patellaris*, *B. procumbens* and *B. webbiana* [12]. *Beta vulgaris* ssp. *maritima* is considered to be the ancestor of sugar beet, and it has been found to be a precious source of resistance against numerous biotic and abiotic stresses [13].

Several diseases and pests reduce the yielding potential of sugar beet crop with infections by viruses and fungi being the major biotic stresses [9, 14, 15]. One of the most problematic viral diseases is virus yellows (VY), which is caused by a complex of aphid-transmitted viruses in single or mixed infections. In Europe, VY is caused by beet mild yellowing virus (BMYV), beet chlorosis virus (BChV) and beet yellows virus (BYV) [16, 17]. BMYV and BChV belong to the family *Solemoviridae*, genus *Polerovirus* and they are mainly transmitted by the green peach aphid (*Myzus persicae*) in a persistent manner [18, 19]. Sugar beet plants infected by BMYV, BChV or BYV show chlorosis in the old leaves from 4 to 6 weeks after virus infection [19]. These chlorotic areas expand until the whole leaf becomes yellow and brittle. The yellowing symptoms of leaves reduce photosynthesis affecting the ability of the plants to grow and resulting in yield loss [16, 19–21]. Neonicotinoids have been used for preventing VY through aphid management, but because of the recent ban on neonicotinoids in Europe, the risks for outbreaks of VY have increased dramatically [22–24]. Moreover, climate change also may increase the risk for VY outbreaks in the future [25].

For sustainable management of VY, it will be important to include the cultivation of resistant or tolerant varieties. There are several partially resistant/tolerant lines of sugar beet against BYV, BChV and beet western yellows virus (BWYV) [19, 26]. To BMYV, partial resistance has been identified in other species belonging to the genus *Beta*, like wild sea beet and also from other cultivated forms of beets like fodder beet, garden beet, and leaf beet, and the trait has been successfully introgressed into sugar beet breeding lines [27, 28]. For another polerovirus, turnip yellows virus (TuYV), several sources of resistance have been identified in *Brassica* species [29]. So far, most of the QTLs reported for TuYV resistance are quantitative, dominantly inherited and with reduction in virus titre [30, 31].

To head towards the development of more tolerant or resistant varieties, knowledge on the host responses to the virus at the transcriptomic level would be highly useful. Transcriptome profiling utilizing high-throughput sequencing (HTS) techniques enables the quantification of transcript levels and comparing the differences in these levels between different conditions. It is then possible to identify putative genes and pathways associated with the defence response to pathogens. Several studies have previously been carried out using transcriptome sequencing to identify candidate genes in sugar beet for resistance and susceptibility to pathogens [10, 32–34].

The different resistance mechanims against viruses in plants obstruct the different steps required for completion of the virus life cycle, like replication, cell-to-cell or long distance movement. RNA silencing is the major defence of plants against viruses, where they recognise and degrade double-stranded (ds) RNA [35]. Plants limit pathogen infections utilising both pattern-triggered immunity (PTI) as well as by recognition of effectors through *R* genes, inducing effector triggered immunity (ETI) [36, 37]. Viral dsRNA can act both as inducer of PTI and RNA interference [37]. Other known defence mechanisms to viruses in plants consist of cellular protein degradation, like the ubiquitin/26S proteasome system (UPS) and the autophagy-mediated degradation pathway, and resistance to virus movement [38–41]. Viral resistance can also be the consequence of mutations in susceptibility genes or in negative regulators of plant defence [42]. In many plant-virus interactions, eukaryotic translation initiation factors (eIFs) are used by viruses for translation and replication, and may then act as susceptibility factors. This has been tested also for poleroviruses where knock-out mutations of eIFs in *Arabidopsis thaliana* resulted in reduced titres of BMYV and BChV [43], while in sugar beet, knock-out of eIFs resulted in reduced titre for BChV, but not for BMYV [44].

We carried out inoculation experiments to compare the response to BMYV by observing symptoms and determining the virus titre in a resistant wild beet and a susceptible genotype of sugar beet. In addition, we conducted RNA sequencing (RNAseq) analyses to identify differentially expressed genes (DEGs) as a response to BMYV infection and elucidate the underlying molecular mechanisms. Candidate genes detected through this study are a valuable resource for future functional validation of target genes and pave the way for a deeper understanding of the interaction between sugar beet and BMYV. The improved understanding of the mechanism of resistance will aid in sugar beet breeding programs to develop resistant varieties.

## Materials and methods

### Experimental setup and virus inoculation

Responses to BMYV infection were studied in two genotypes of beet: a BMYV-resistant genotype of wild beet (A) and a susceptible breeding line of sugar beet (B) from the germplasm collection of DLF Beet Seed, Landskrona, Sweden. Compared to the susceptible genotype, plants of the resistant genotype exhibited rosetted and thicker leaves, but no significant differences in growth traits were observed. The experiment was carried out at the quarantine facility of DLF Beet Seed in Landskrona, Sweden with a temperature of 22 °C during the day and 20 °C during the night, and a light duration of 16 h. The germinations were synchronized between the genotypes and after germination, each seedling was placed in a separate pot. The seedlings were grown in soil specifically made for DLF Beet Seed (Emmaljunga Torvmull, Sweden). Viruliferous *M. persicae* aphids and healthy aphids were maintained in different chambers within the quarantine facility before application to plants. Four plant treatments were used: plants exposed to *M. persicae* aphids carrying BMYV (Inoculated) or aphids without BMYV (Healthy), plants without exposure to aphids or BMYV (Non-inoculated) and plants treated with only insecticide (Insecticide control). Within each treatment, plants of the two genotypes were randomised for observation of symptoms and sampling at 0, 1, 4, 14, 21 and 28 days post-inoculation (DPI). There were six biological replicates for each time point and treatment (Additional file 1). For each inoculated plant, 10 viruliferous *M. persicae* aphids were transferred to the first true leaf and at 4 DPI, the aphids were killed by application of an insecticide (Teppeki, Nordic Alkali AB, Sweden). Tables containing plant trays were covered with sticky foil to restrict aphid movement and covered with fleece to avoid cross-contamination between treatments (Additional file 1).

### Collection of leaf samples

For virus testing and RNA extractions, inoculated leaves (referred also to as old leaves) from 0, 1, 4, 14, 21 and 28 DPI as well as systemic leaves (referred also to as young leaves) from 14, 21 and 28 DPI were collected and frozen in liquid nitrogen for subsequent storage at −80 °C (Additional file 7). The leaf samples were homogenized using a high-speed benchtop tissue homogenizer (Fast-Prep^®^−24, Classic, MP Biomedicals, USA) at 5.0 m/s for 3 × 60 s. Homogenized leaf tissue (100 mg per sample) was transferred to a 1.5 ml microcentrifuge tube for RNA extraction.

### Virus detection by ELISA and RT-PCR

Triple antibody sandwich enzyme-linked immunosorbent assay (TAS-ELISA) and RT-PCR were carried out on the collected leaf samples for ensuring that there was no cross contamination and to check the robustness of the inoculation using viruliferous aphids. For leaf samples collected at 14, 21 and 28 DPI, TAS-ELISA for BWYV (Deutsche Sammlung von Mikroorganismen und Zellkulturen) was used for virus detection following the manufacturer’s protocol [25], while leaf samples collected at 0, 1 and 4 DPI were too small for reliable ELISA testing. RNA extracts of all leaf samples (see section below) were tested for BMYV using RT-PCR and universal primers for the coat protein (CP) gene of poleroviruses [25, 45].

### RNA extraction and sequencing

RNA was extracted from sugar beet leaves using RNAqueous^TM^- 4PCR total RNA Isolation Kit (Invitrogen), as per manufacturer’s guidelines. The RNA concentration and purity were determined with a NanoDrop ND-1000 (Thermo Fisher Scientific, MA, USA). Agilent 2100 Bioanalyzer (Agilent Technologies, CA, USA) was used to estimate the RNA integrity number (RIN) and all RNA extracts with a RIN value above 8.0 were used for library preparation. Sequencing libraries were prepared from 500 ng total RNA using the TruSeq stranded mRNA library preparation kit (Illumina Inc., CA, USA) according to the manufacturer’s protocol, including poly-A selection and using unique dual indexes. Sequencing was carried out by the SNP&SEQ Technology Platform in Uppsala with Illumina NovaSeq 6000 platform, S4 flowcell and v1.5 sequencing chemistry. Paired-end 150 bp mRNA reads were generated for all 120 samples. Raw sequencing data obtained in this study have been deposited in National Centre for Biotechnological Information (NCBI) under the Bioproject accession number PRJEB80223.

### Quantification of BMYV by RT-qPCR

For absolute quantification of BMYV, RT-qPCR was run with three biological replicates per treatment, where each replicate represents an individual plant. The cDNA was synthesised from 1 µg total RNA using Maxima First Strand cDNA Synthesis Kit (Thermo Fisher Scientific, Lithuania) according to the manufacturer’s protocol. For RT-qPCR analysis, 4 µl of 10-fold diluted cDNA and Maxima SYBR Green/ROX qPCR Master Mix (2X) (Thermo Fisher Scientific, Lithuania) were used following the manufacturer’s guidelines. The primers used for qPCR were 5’ AAAGTTATGAGCGGTAGGCAA 3’ (F) and 5’ CACTTGTTCGTTGAACCGACT 3’ (R), 0.3 µM each, with an amplicon length of 90 bp, targeting the CP gene of the virus. Thermal cycling conditions were initial incubation at 95 °C for 3 min followed by 39 cycles of 10 s at 95 °C, 30 s at 60 °C, 30 s at 72 °C, and a final denaturation at 95 °C for 5 s. The qPCR was followed by melt curve analysis at 65–95 °C, with an increment of 0.5 °C/5 s. A standard curve for absolute quantification was obtained with qPCR using 4 µl of 10-fold serial dilutions with 100 to 0.01 pg µl^−1^ of plasmid pJET1.2 containing the CP gene of BMYV [25]. Data analysis to calculate the virus copy numbers was executed using the BioRad CFX Manager Version 3.1 application software and with the formula: number of molecules = (ng of dsDNA of template plasmid) × (6.0233 × 10^23^/mol) × (1/number of bases) × (1/660 g/mol) × (1/10^9^ ng/g) [46]. The calculations were based on three biological and three technical replicates and the average copy number was calculated for each time point. A t-test assuming unequal variance was performed to identify significant differences (P-value ≤ 0.05) in virus copy number between the genotypes at different time points.

### Read processing and differential gene expression analysis

The aim of the transcriptome profiling was to compare the response to BMYV infection in a resistant wild beet and a susceptible sugar beet genotype, and identify the differentially expressed genes (DEGs) to understand the resistance mechanism. RNAseq data was bioinformatically analysed using the nf-core/rnaseq pipeline (v. 3.10.1) [47] to prepare quantified expression matrices. The analysis was conducted on an UPPMAX high-performance computing cluster using singularity for reproducibility (https://github.com/abu85/rna_seq_analysis_sugarbeet*).* In brief, a quality control was performed on the generated paired-end mRNA reads with FastQC v0.11.9 [48]. Adapter and low-quality base trimming was performed using wrapper Trim Galore v. 0.6.7 [49]. Ribosomal reads were removed with the SortMeRNA v.4.3.4 option [50] and the default rRNA database. Pseudoalignment and transcript quantification were performed on cleaned reads using Salmon v.1.9.0 [51] providing the index generated from the transcriptome of the sugar beet reference genome EL10 [4].

The quantified transcript abundances were imported into the R environment [R version 4.3.1, accessed on 2023-06−16 ucrt] using the package tximport v.1.28.0 [52]. Principle component analysis (PCA) was performed using R package DESeq2 v.1.40.1 after variance stabilizing transformation of the data [53]. Two-dimensional PCA plots were developed using ggplot2 v.3.4.3 [54]. To compare transcript levels of inoculated and healthy plants at the same time point and leaf age, differential gene expression analysis was performed using DESeq2 v1.40.1 [53]. In DESeq2, the Wald test was used for hypothesis testing, where P value was set to 0.05 and the P values obtained were corrected by multiple testing using the Benjamini and Hochberg method by default to reduce false positives [55]. Genes with a false discovery rate (FDR) value < 0.05 and log2 fold change >1 or < −1 were considered significant. Venn diagrams were created and visualised using an online webtool (https://bioinformatics.psb.ugent.be/webtools/Venn/) to assess overlap between different experimental groups. Data obtained after DESeq2 analysis for each genotype, time point and leaf age was analysed separately. The transcript levels were compared between inoculated and healthy samples where the levels of healthy samples were kept constant resulting in obtaining the significantly upregulated and downregulated genes in response to BMYV infection. Similarly, transcript levels were compared between insecticide-treated and healthy samples in order to identify genes with significant differential expression in response to the insecticide treatment. The genes significantly up- or down-regulated specifically in response to insecticide treatment were not considered for the gene ontology analysis.

### Analyses of gene ontology and KEGG pathways

Many genes in the EL10.1 sugar beet reference genome have not been functionally annotated. Hence, gene IDs of *Arabidopsis thaliana* corresponding to the translated protein sequences of genes from the sugar beet reference genome EL10.1 were obtained from the *A. thaliana* database TAIR10, released 2019-07-11, using BLASTp [10]. The *A. thaliana* gene IDs obtained were used for generating the annotations in R with the Annotationhub bioconductor package [56] followed by GO enrichment analysis with the clusterProfiler package [57]. The *A. thaliana* annotations were used to identify the transcription factor families in the *A. thaliana* database TAIR10. KEGG pathway analyses were performed using ShinyGO v.077 [58] to identify the most significantly enriched pathways (FDR < 0.05, calculated based on nominal p-value from the hypergeometric test), with *A. thaliana* as reference species [10]. The enriched GO IDs were summarised and the interacting biological processes were visualized using REVIGO webtool [59].

## Results

### Response to BMYV infection in resistant and susceptible plants

**Fig. 1:**
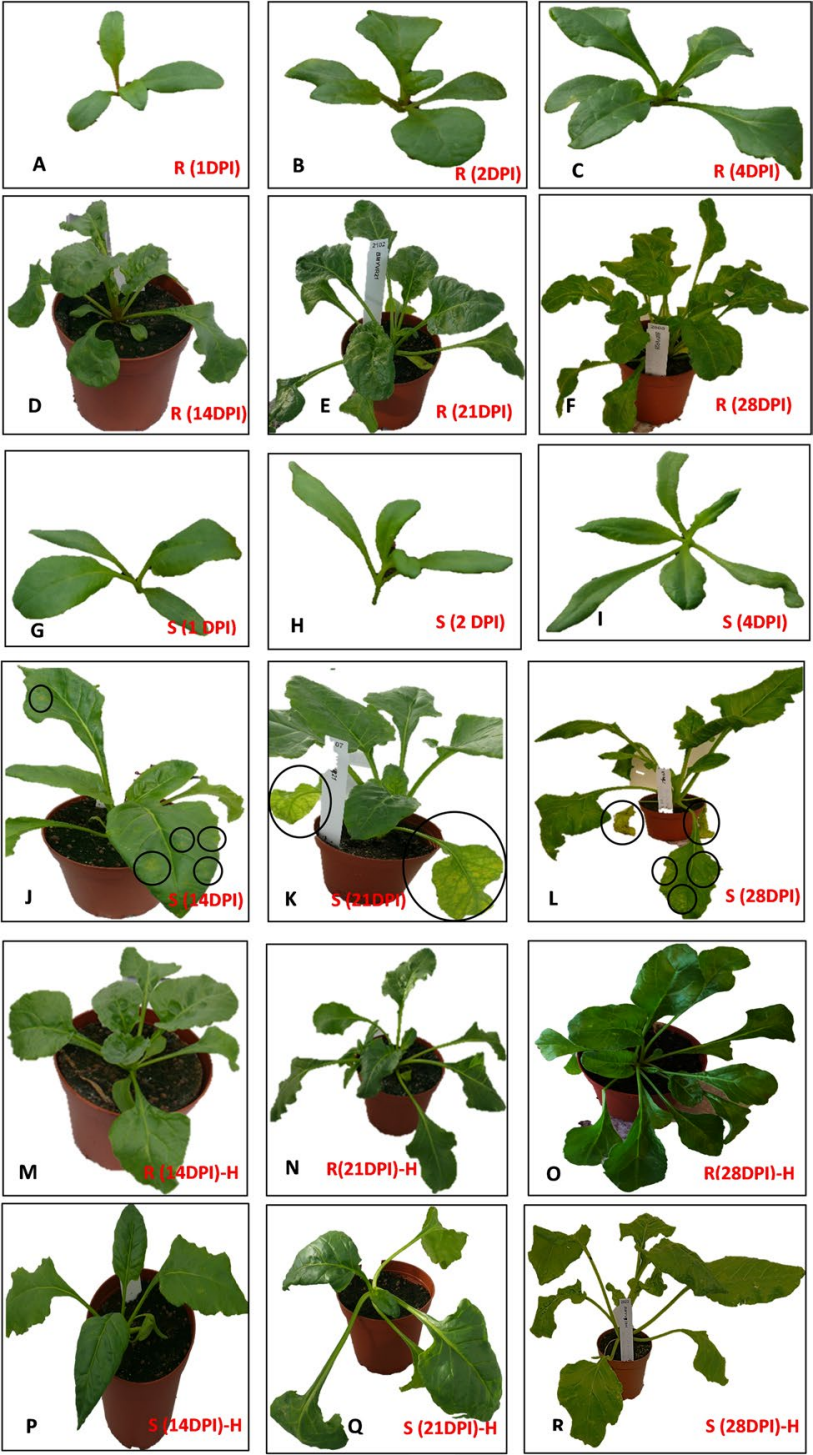
Response to BMYV infection in plants of a resistant genotype of beet and a susceptible genotype of sugar beet. **A**-**F** BMYV-inoculated resistant plants at 1, 2, 4, 14, 21 and 28 DPI (R); **G**-**L** BMYV-inoculated susceptible plants at 1, 2, 4, 14, 21 and 28 DPI (S). **M**-**O** Healthy resistant plants at 14, 21 and 28 DPI (H). **P**-**R** Healthy susceptible plants at 14, 21 and 28 DPI (H). The circles show areas of leaves with chlorotic symptoms

In susceptible plants, small speckles of chlorotic symptoms began to appear in BMYV-inoculated leaves at 14 DPI and subsequently progressed to the entire leaf, which turned chlorotic at 21 DPI. At 28 DPI, the inoculated leaves of susceptible plants had turned completely yellow and were found to be dead, while virus symptoms started to emerge in the second leaf pair. In comparison, plants of the resistant genotype stayed green without showing any symptoms of virus infection during the complete experiment (Fig. 1). The insecticide treatment did not result in any visible effects on the plant.

**Fig. 2:**
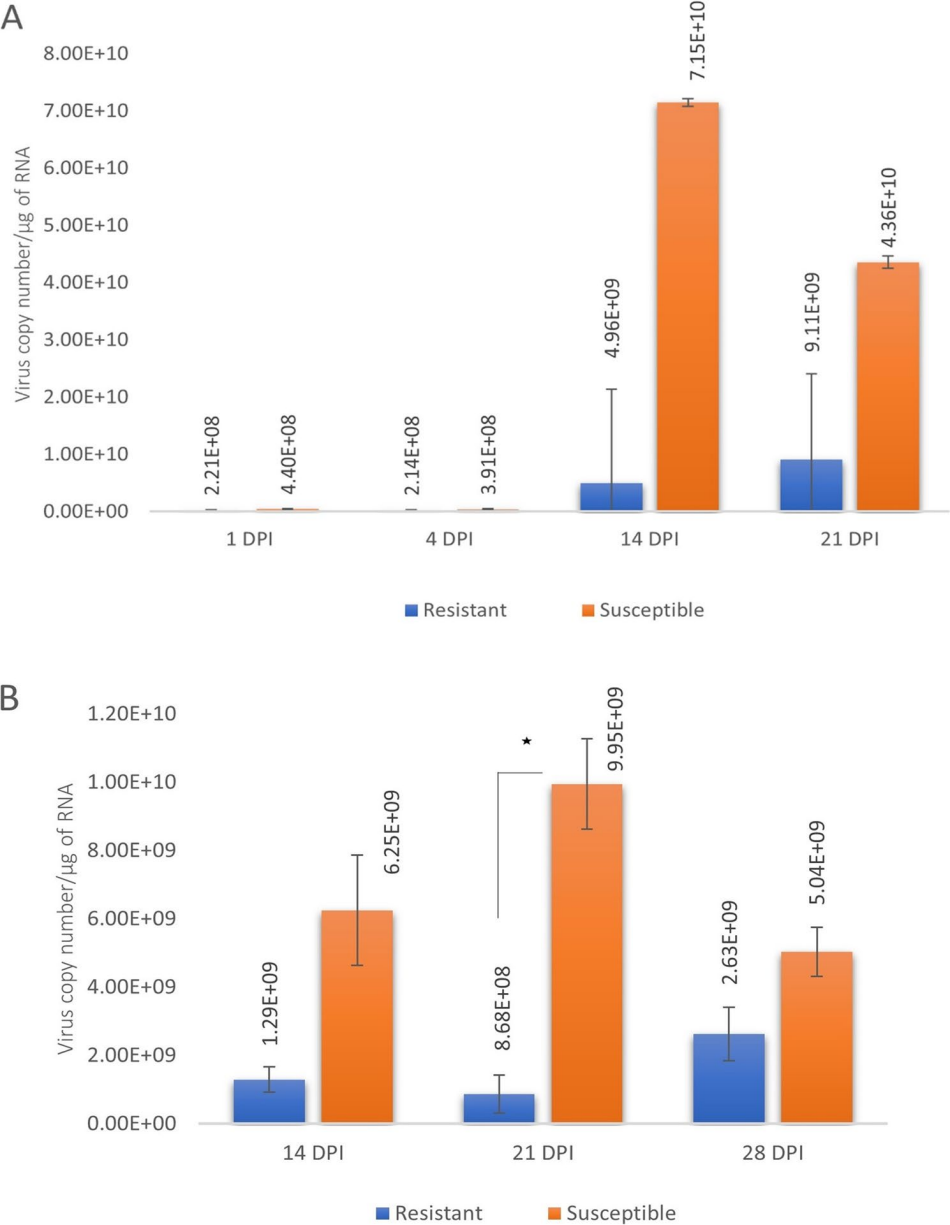
Copy number of BMYV as determined by RT-qPCR in 1 µg RNA of leaves collected at different time points after inoculation. **A** old (inoculated) leaves, **B** young (systemic) leaves. Asterisk represents significant difference in BMYV copy number between the genotypes (P-value ≤ 0.05) by t-test assuming unequal variance

Using RT-qPCR, a low level of BMYV was detected already at 1 DPI in inoculated leaves of both susceptible and resistant plants (Fig. 2A). The virus level remained low at 4 DPI, but had increased at 14 DPI. At 14, 21 and 28 DPI, the virus level was always higher in the susceptible plants compared to the resistant ones in both old (inoculated) and young (systemic) leaves, ranging from 2-fold to 14-fold difference at different time points (Fig. 2A and B). However, a significant difference (p-value ≤ 0.05) in viral load between genotypes was observed only at 21 DPI in young leaves (Fig. 2B). The temporal variation in virus titre also differed between the two genotypes. The old leaves in the resistant genotype displayed a gradual increase in virus titre at 14 and 21 DPI, whereas in the susceptible genotype, the virus titre was highest at 14 DPI and then decreased by 21 DPI (Fig. 2A). In young leaves of resistant plants, the virus titre was similar at 14 DPI and 21 DPI, and then increased at 28 DPI, whereas in young leaves of suceptible plants, the virus titre increased at 21 DPI compared to 14 DPI and then decreased at 28 DPI (Fig. 2B). For both genotypes, the virus titre was lower in young leaves than old leaves at 14 and 21 DPI (Fig. 2A and B).

### Transcriptomic sequencing and principal component analysis

To understand the beet responses to BMYV infection, the transcript data obtained by RNAseq for inoculated and healthy leaf samples from old and young leaves of both genotypes was compared. On average, the sequencing yielded between 27.3 and 82.7 million raw reads (151 bp) per sample with a GC content of 43–46%. After quality check and removal of adaptor sequences, the average number of reads processed for both genotypes was 34 million. The average number of reads mapped to the EL10 reference genome of sugar beet was 27 million (75–81%) for the susceptible genotype and 26 million (75–79%) for the resistant genotype (Additional file 2). A very similar percentage of mapping for both genotypes indicates that only a limited genotype bias was introduced by using the EL10 sugar beet reference genome for resistant as well as susceptible genotype. Reads for BMYV were identified for inoculated leaves (0–0.25%), even if the BMYV genome does not have a poly-A-tail, but not for non-inoculated or insecticide-treated leaves.

In order to understand the patterns of similarity between the different samples in response to BMYV infection based on the differential gene expression, PCA was performed using the RNAseq data (Additional file 3). BMYV inoculation did not show as a major source of variation. The analysis revealed that for both inoculated (old) leaves and systemic (young) leaves, there was clustering based on genotype. For inoculated leaves, there was a separation between samples collected at early time points after inoculation (0, 1 and 4 DPI) and those collected later (14 and 21 DPI)(Additional file 3 A). For systemic leaves, there was an overlap between time points, e.g., for leaves of resistant plants collected at 21 and 28 DPI as well as for those collected at 14 and 21 DPI (Additional file 3B).

### Differentially expressed genes in response to BMYV infection

Out of 24,208 genes from the reference genome, it was possible to assemble 21,042 genes from the RNAseq data for the resistant genotype and 20,454 genes for the susceptible genotype, combining samples across all time points and leaf ages. In response to BMYV infection, a total of 198 genes were differentially expressed in the resistant genotype, whereas 1218 genes were differentially expressed in the susceptible genotype. DEGs identified when comparing insecticide-treated plants with the plants exposed to aphids without virus (healthy) were removed from the analyses. A higher number of significant DEGs (FDR < 0.05 and log2FoldChange > 1 or < −1) were identified for the susceptible genotype than the resistant genotype when comparing inoculated and healthy samples (Table 1). Among the significant DEGs as a response to BMYV infection, 137 genes were unique to the resistant genotype and 1,157 genes were unique to the susceptible genotype. The level of differential expression was also higher for the susceptible genotype compared to the resistant genotype. This indicates that the response to BMYV infection at the molecular level is much stronger in the susceptible genotype.

**Table 1:** Summary of DEGs significantly (FDR < 0.05 and log2FoldChange > 1 or < −1) upregulated or downregulated in response to BMYV inoculation in old and young leaves at different time points in the resistant and susceptible genotype

Leaf age	Time point	DEGs			
	(DPI)	Resistant		Susceptible	
		Up	Down	Up	Down
Old	1	0	0	0	0
	4	1	3	0	3
	14	4	2	301	303
	21	16	27	257	455
Young	14	16	5	16	5
	21	1	0	0	1
	28	43	117	23	34

The initial transcriptional response to BMYV infection was weak in both susceptible and resistant plants. At 1 DPI, no DEGs were identifed and only a few at 4 DPI (Table 1). The inoculated leaves of susceptible plants then showed a drastic increase of DEGs at 14 and 21 DPI with 604 and 702 DEGs, respectively. In resistant plants, there was only a minor increase of DEGs at the corresponding time points. For young leaves, the number of significant DEGs was the same (16 upregulated and 5 downregulated DEGs) at 14 DPI for both genotypes followed by only one DEG at 21 DPI for each genotype. At 28 DPI, there was an increase in DEGs for both susceptible and resistant plants, but the number of DEGs was higher for resistant (160 DEGs) than for susceptible plants (57 DEGs).

**Fig. 3:**
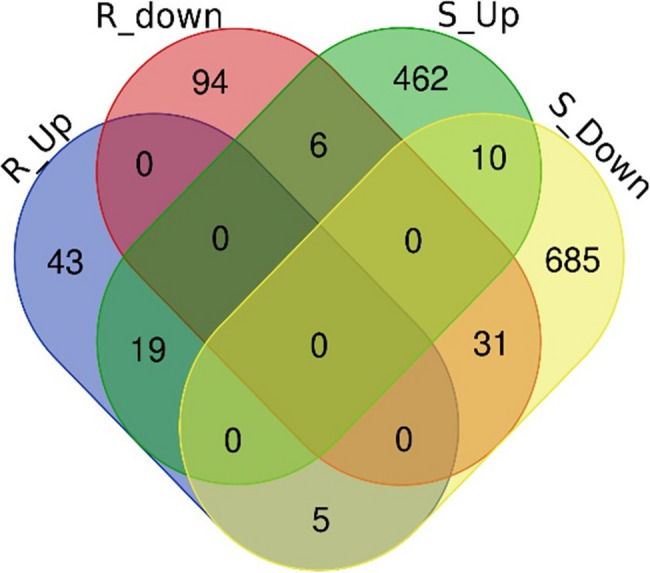
Venn diagram showing differentially expressed genes (FDR < 0.05, log2FoldChange > 1 or < −1) unique or shared between resistant (R) and susceptible (S) genotypes in response to BMYV infection combining time points as well as old and young leaves. Up and Down indicate genes that were up- or downregulated, respectively, in response to BMYV infection

**Fig. 4:**
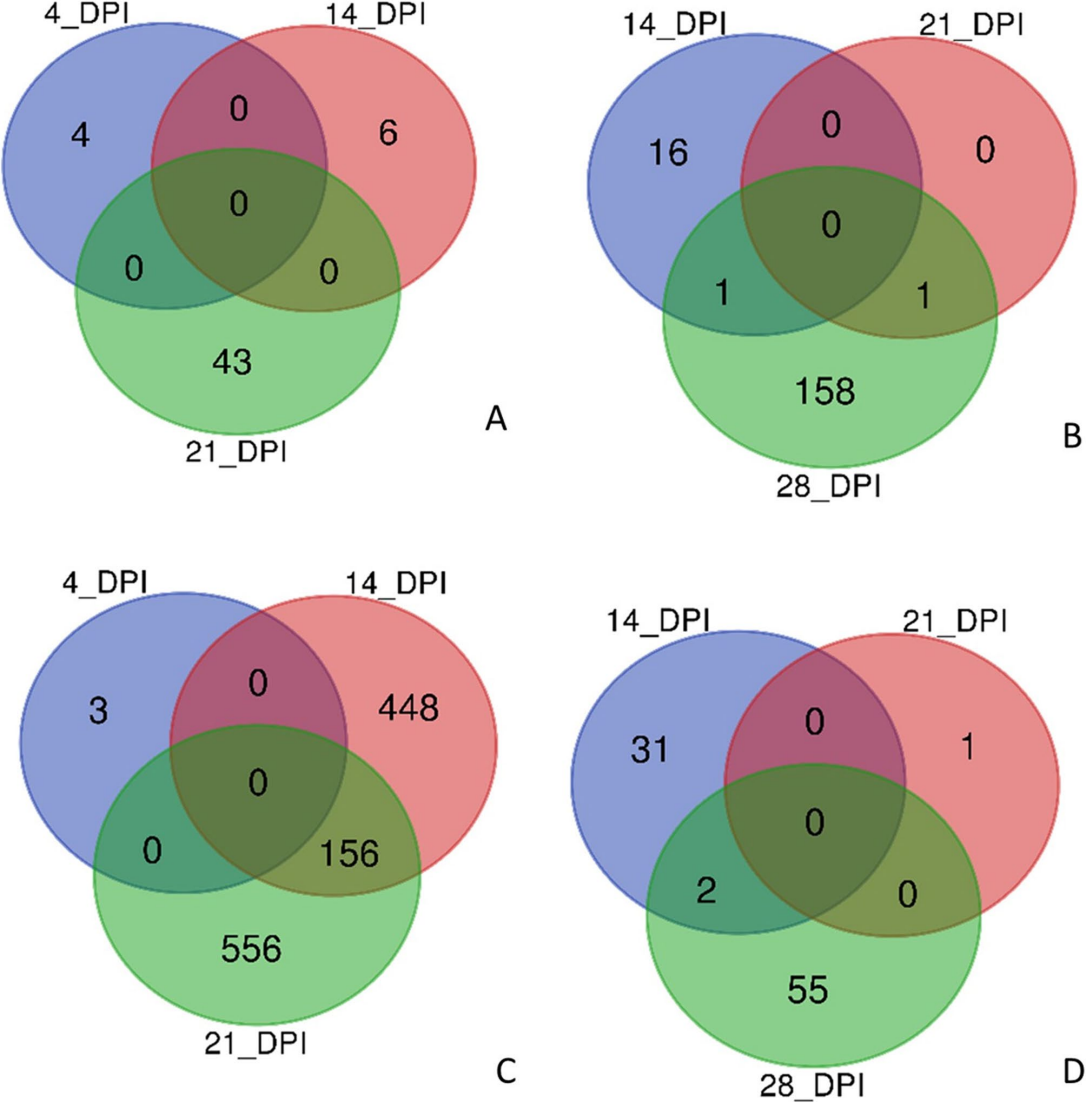
Venn diagrams depicting differentially expressed genes in response to BMYV infection (FDR < 0.05, log2FoldChange > 1 or < −1) that are unique or shared between different time points in (**A**) old leaves of resistant plants, (**B**) young leaves of resistant plants, (**C**) old leaves of susceptible plants and (**D**) young leaves of susceptible plants

Among the significant DEGs, 43 of them were upregulated only in the resistant genotype, and 462 only in the susceptible, while 5 DEGs were upregulated in resistant plants and downregulated in susceptible plants (Fig. 3; Additional file 8). For old leaves of susceptible plants, 156 DEGs were shared between 14 and 21 DPI, but this was not the case for resistant plants (Fig. 4A and C). For young leaves, only few genes were shared between time points for both genotypes (Fig. 4B and D). The change in transcript levels as a response to BMYV infection was larger in susceptible than in resistant plants (Additional files 9 and 10) where only few genes were strongly affected (log2FoldChange > 1 or < −1).

The DEGs of the resistant genotype in response to BMYV infection (Additional file 10) included multiple genes, which are associated with biotic stress response in plants. Fourteen upregulated genes were specifically identified as likely to be involved in resistance responses to BMYV based on their known role in plant immune responses, virus accumulation in the host and symptom severity (Table 2). Seven genes significantly upregulated in the resistant genotype encode proteins involved in protein processing in the endoplasmic reticulum (ER) and these genes were not significantly differentially expressed in the susceptible genotype: EL10Ac6g15151 (Leucine-zipper of ternary complex factor MIP1), EL10Ac3g07108 (EGF domain-specific O-linked N-acetylglucosamine transferase), EL10Ac3g06084 (Probable E3 ubiquitin ligase SUD1), EL10Ac8g20617 (Vacuolar protein sorting-associated protein 28), EL10Ac5g12002 (Probable xyloglucan endotransglucosylase/hydrolase protein 30), EL10Ac3g05286 (Calcineurin B-like protein 4) and EL10Ac4g09930 (Luminal-binding protein 4). Other genes putatively linked to resistance include EL10Ac3g07016 (Putative disease resistance protein RGA3), EL10Ac4g07519 (Probable LRR receptor-like serine/threonine-protein kinase At4g26540), EL10Ac1g00851 (Chalcone synthase), EL10Ac6g13175 (Homeobox-leucine zipper protein ATHB-13), EL10Ac6g13275 (Protein ENHANCED DISEASE RESISTANCE 2-like), EL10Ac3g07017 (Putative disease resistance protein RGA4), EL10Ac6g13345 (Beta-glucosidase 12) and EL10Ac5g10982 (Transcription factor TCP15). Interestingly, the chloroplastic gene EL10Ac5g11039 (Thioredoxin-like 1–2) was one among the five genes that was upregulated in the resistant genotype, but downregulated in the susceptible genotype. This DEG was shared between young leaves collected at 21 and 28 DPI from resistant plants.

**Table 2:** List of selected uniquely upregulated genes in the resistant genotype involved in viral protein degradation pathways, plant defence responses to viruses and in symptom development

EL10 gene ID	Sugar beet annotation	Description
EL10Ac5g11039^a^	Thioredoxin-like 1–2, chloroplastic	Role in preventing oxidative damage of antioxidant enzymes, reducing virus accumulation in the host plant, SA-mediated defence responses
EL10Ac3g07016	Putative disease resistance protein RGA3	Disease resistance protein (CC-NBS-LRR class) family involved in plant defence to pathogens
EL10Ac3g07017	Putative disease resistance protein RGA4	
EL10Ac2g03638	Cytochrome P450 CYP73A100	Role in secondary metabolite production and in response to wounding
EL10Ac8g18590	Cytochrome P450 734A1	
EL10Ac6g15151	Leucine-zipper of ternary complex factor MIP1	Involved in endoplasmic-reticulum associated protein degradation (ERAD) pathway
EL10Ac4g09930	Luminal-binding protein 4	
EL10Ac3g07108	EGF domain-specific O-linked N-acetylglucosamine transferase	
EL10Ac3g06084	Probable E3 ubiquitin ligase SUD1	
EL10Ac8g20617	Vacuolar protein sorting-associated protein 28 homolog 2	
EL10Ac3g05286	Calcineurin B-like protein 4	
EL10Ac4g07519	Probable LRR receptor-like serine/threonine-protein kinase At4g26540	Role in recognition of PAMPs and triggering immune respones
EL10Ac1g00851	Chalcone synthase	Role in secondary metabolite production
EL10Ac5g10982	Transcription factor TCP15	Transcription factor family involved in ETI

^a^Upregulated gene in resistant and downregulated in susceptible genotype

### Transcription factor genes differentially expressed in response to BMYV infection

**Fig. 5:**
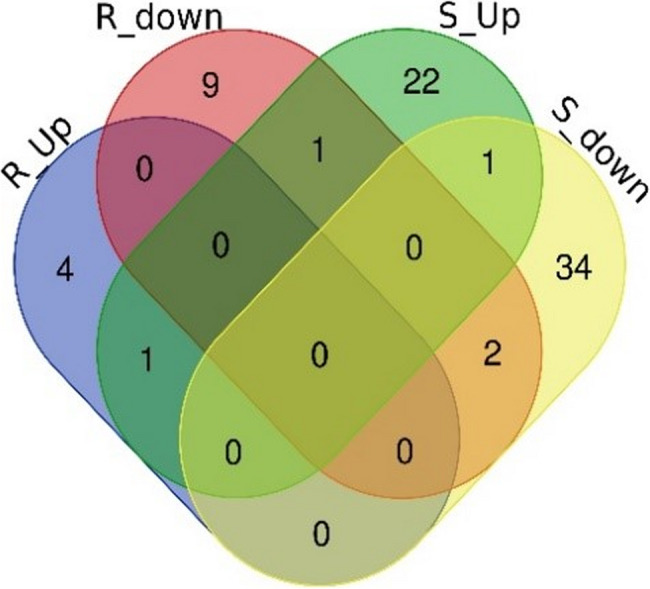
Total number of transcription factor genes uniquely expressed or shared between the resistant (R) and susceptible (S) genotypes in response to BMYV infection combining all the time points and leaf ages. Up and Down indicate genes that were up- or downregulated, respectively, in response to BMYV infection

Among the significant DEGs in response to BMYV infection, 74 transcription factor (TF) genes were identified. In the resistant plants, 4 TF genes were uniquely upregulated and 9 TF genes downregulated, whereas in the susceptible plants, 22 TF genes were uniquely upregulated and 34 TF genes downregulated (Fig. 5). The TF gene families found to be differentially expressed in response to BMYV infection in the resistant genotype and known to be associated with immune responses to pathogens in plants include bHLH, AP2-EREBP, MYB and NAC (Additional file 11).

### Gene ontology analysis and KEGG pathways

Gene ontology analysis was carried out to obtain insights into the biological, cellular and molecular processes underlying responses to BMYV infection in leaves of resistant and susceptible genotypes. The analysis was performed with all significant DEGs using annotations from *A. thaliana*, and it was carried out separately for each time point and for inoculated (old) and systemic (young) leaves.

**Fig. 6:**
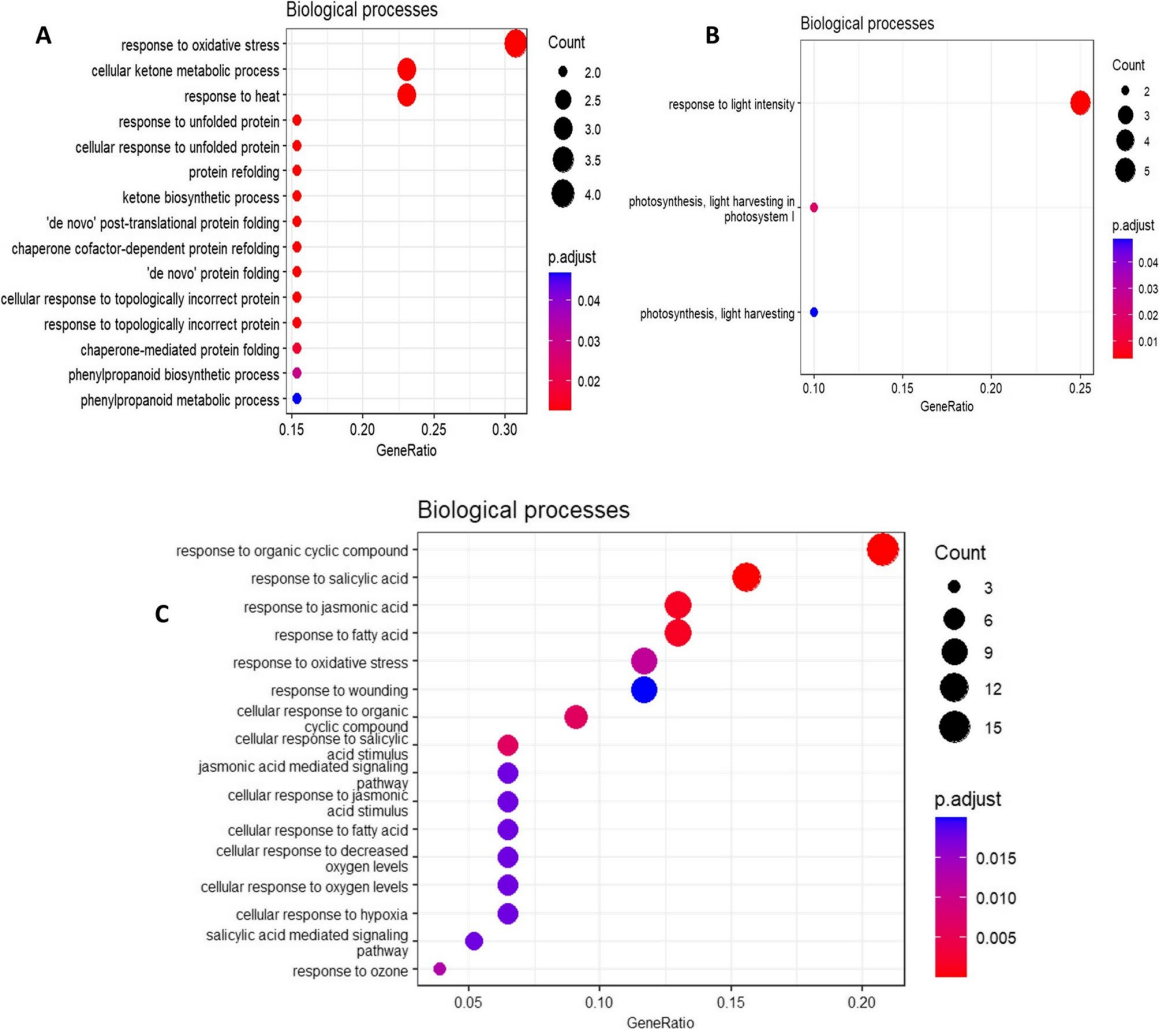
Biological processes upregulated (**A**) and downregulated (**B**) in old leaves at 21 DPI and (**C**) downregulated pathways in young leaves at 28 DPI in the resistant genotype in response to BMYV infection (P adj < 0.05)

For the resistant genotype, no enriched biological processes were identified for inoculated leaves collected at 1, 4 or 14 DPI, while at 21 DPI, 15 biological processes were upregulated in inoculated leaves with response to oxidative stress (GO:0006979) as the most upregulated one (Fig. 6A, Additional file 12). Eleven of the GO IDs were related to the biological response of protein folding and unfolding represented by 18 gene counts (Additional file 12). The molecular processes upregulated at this time point included four GO IDs related to the same biological processes: misfolded protein binding (GO:0051787), protein folding chaperone (GO:0044183), heat shock protein binding (GO:0031072) and unfolded protein binding (GO:0051082) (Additional file 12). Phenylpropanoid biosynthetic and metabolic processes (GO:0009699, GO:0009698) were also among the upregulated biological processes at this time point (Fig. 6A). At 21 DPI, response to light intensity (GO:0009642) and photosynthesis-related pathways (GO:0009768, GO:0009765) were among the downregulated biological processes in inoculated leaves of resistant plants (Fig. 6B). In systemic leaves at 28 DPI, response to organic cyclic compunds (GO:0014070), response to salicylic acid (SA; GO:0009751) and response to jasmonic acid (JA; GO:0009753) were the top biological processes found to be downregulated (Fig. 6C).

**Fig. 7:**
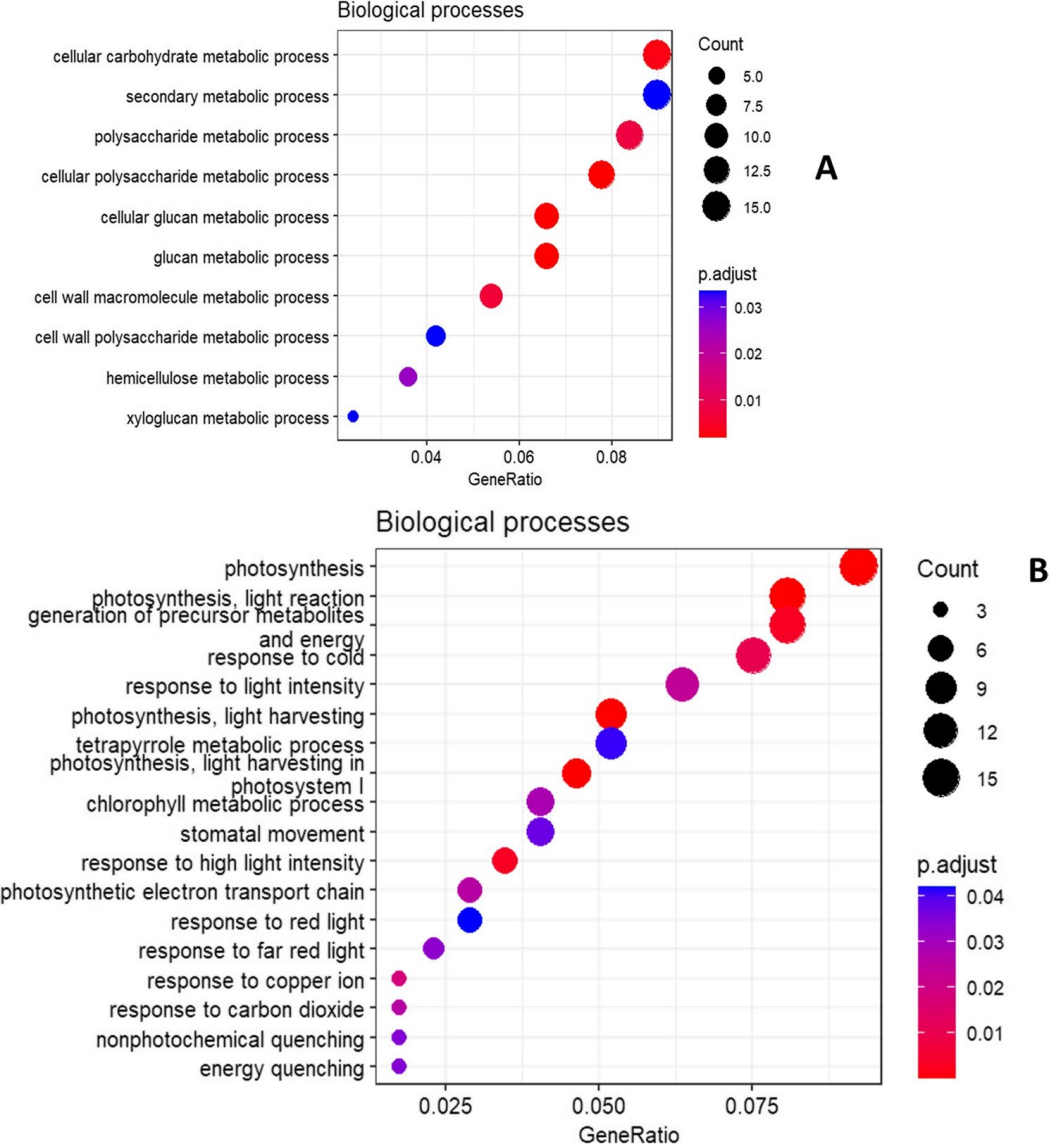
Biological processes upregulated (**A**) and downregulated (**B**) at 14 DPI in the susceptible genotype in response to BMYV infection in old leaves (P adj value < 0.05)

In the susceptible genotype, enriched biological processes were found for inoculated leaves collected at 14 DPI and 21 DPI as well as for systemic leaves from 14 DPI and 28 DPI (Additional file 13). In inoculated leaves at 14 DPI, cellular carbohydrate metabolic process (GO:0044262) and secondary metabolic process (GO:0019748) were the top upregulated biological processes with 15 gene counts each (Fig. 7A). At the same time point, four biological processes related to photosynthesis were downregulated with 47 genes (Fig. 7B). For inoculated leaves collected at 21 DPI, many biological processes related to DNA damage response and DNA synthesis were upregulated, among which the top processes include DNA metabolic process (GO:0006259), cellular response to DNA damage stimulus (GO:0006974) and DNA repair (GO:0006281) (Additional file 4). No KEGG pathways were enriched for differentially expressed genes in the resistant genotype whereas in the susceptible genotype, photosynthesis, DNA replication and biosynthesis of secondary metabolites were significantly enriched processes (Additional file 5). The representative GO IDs for both genotypes obtained using the REVIGO web tool and visualization in an interactive graph indicated that stress-responsive biological processes are highly linked in the resistant genotype (Additional file 6 A). For the susceptible genotype, the interacting representative GO IDs were mostly related to stress damage response and photosynthesis (Additional file 6B).

## Discussion

In this study, we looked at the early responses of beet to BMYV, quantified the virus level to establish the genotype effect on virus accumulation and further narrowed down to the transcript level changes to understand the responses leading to resistance or disease development. No symptoms were observed in the resistant genotype throughout the 28 days after inoculation whereas in the susceptible genotype, visible symptoms were observed from 14 DPI. Virus titre quantification using RT-qPCR revealed that it was higher in susceptible plants compared to resistant plants at 14, 21 and 28 DPI. When comparing the virus titre between leaves at 14 and 21 DPI, it was found to be lower in young than old leaves for both genotypes. A possible explanation could be that the resistant genotype may pose restrictions to virus movement and thus the virus is accumulating less in the younger leaves in comparison to the susceptible genotype. The difference could also be due to higher virus multiplication rate in susceptible plants. Absence of symptoms and lower virus titre in the resistant genotype indicate that it is neither a complete resistance nor tolerance, but a partial resistance against the virus, similar to what has been found before for BMYV in genotypes of *Beta* spp [27, 28].

Comparisons of the number of significant DEGs in old and young leaves at different time points reveal that there were more DEGs for the susceptible genotype compared to the resistant genotype. This is because plants of the susceptible genotype were more affected by the virus infection showing a stronger response compared to resistant plants. For the resistant genotype, DEGs were more abundant at later time points (21 and 28 DPI), while for the susceptible genotype, there were more DEGs already at 14 DPI. This indicates that plant responses to virus infection appear earlier for the susceptible compared to the resistant genotype. In other transcriptomic studies involving plant response to virus infections, similar to the transcript response in this study, a lower number of DEGs was found for virus-resistant varieties and hosts with less symptoms [60, 61].

**Fig. 8:**
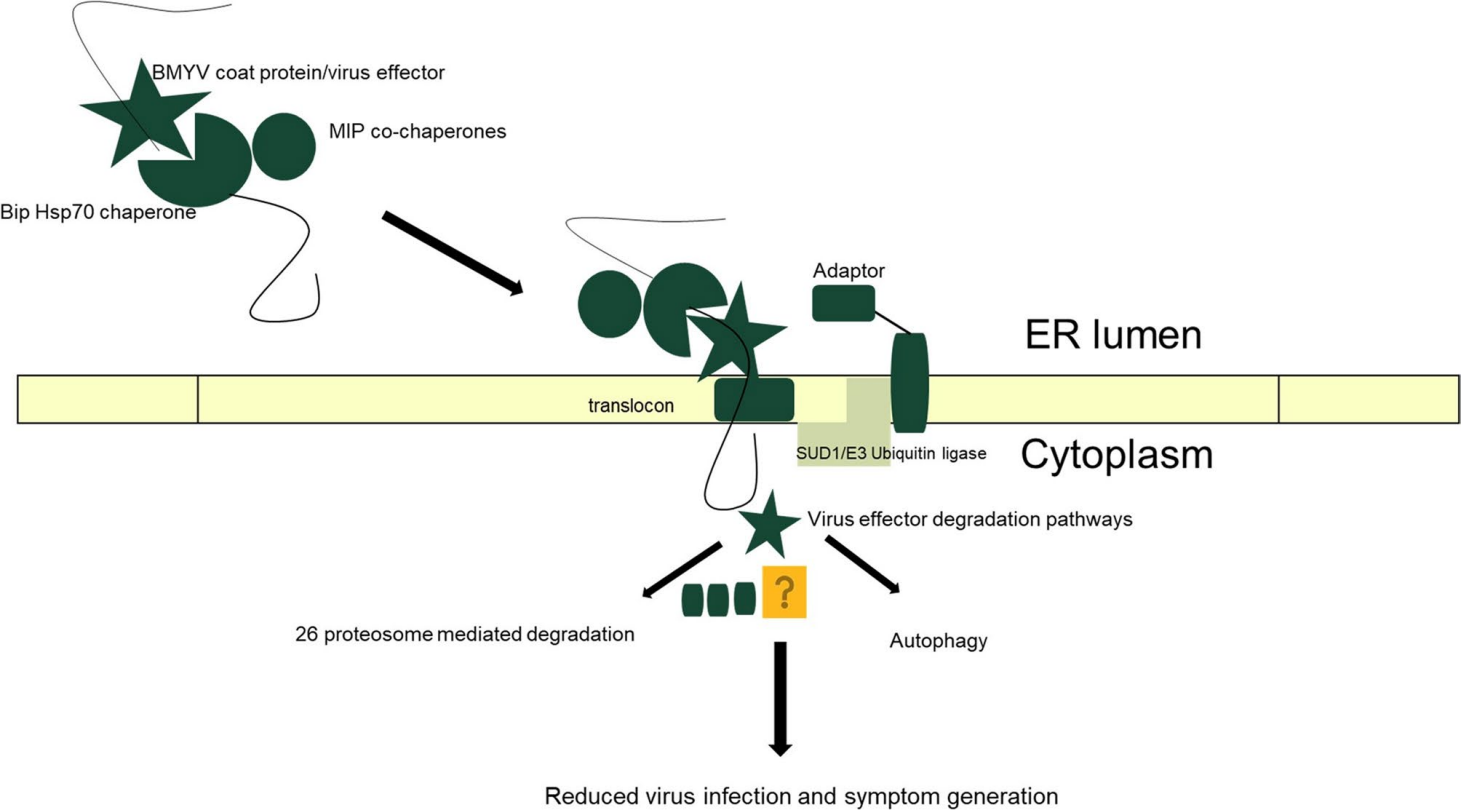
A possible interaction model of BMYV coat protein (CP) and BiP as a host factor. The ERAD machinery in plants is essential for healthy cells. It facilitates removal of malformed or unfolded proteins from the endoplasmic reticulum (ER) and transport into the cytoplasm where they are degraded to reduce ER stress. The foremost step in the ERAD machinery is substrate selection where the BMYV CP gets docked into the substrate-binding domain of the Hsp70 chaperone BiP. The ERAD substrate is transferred to the cytoplasm via a translocon across the ER membrane. The adaptor proteins present in the ER aid in recognition of the substrate (viral CP) by the SUD1/E3 ubiquitin ligase complex. Once the substrate is in the cytoplasm, the viral CP is degraded by the 26S proteosome system or autophagy. This leads to decreased accumulation of viral proteins in the host cells and hence reduced ER stress

Some of the genes significantly upregulated in the resistant genotype encode proteins involved in protein processing in the ER. Protein processing in the ER could be one mechanism contributing to resistance by reducing ER stress. A study carried out to identify host proteins interacting with potato leaf roll virus (PLRV; genus *Polerovirus*) showed that protein processing in ER was one among the GO biological processes enriched in the PLRV-host protein interactome [62]. ER stress is triggered upon virus infection in plants and it needs to be alleviated to prevent cell death [63]. ER stress is detected in plants by transmembrane proteins, which initiate the unfolded protein response (UPR) to restore protein folding in ER and prevent accumulation of malformed or unfolded proteins hence reducing ER stress [64]. This later on leads to expression of the UPR genes encoding calcium-dependent protein chaperones, e.g., Luminal binding protein (BiP), calreticulin, calcineurin, and calmodulin [63–66]. ER-associated degradation (ERAD) pathway, a quality control mechanism in plants, is triggered as a part of the UPR. This machinery functions by translocating the unfolded or misfolded proteins into the cytoplasm, where they are degraded by the ubiquitin/26S proteasome system (UPS) or by autophagy. In this study, out of 43 uniquely upregulated DEGs in the resistant genotype in response to BMYV infection, six of them encode proteins known to be associated with the ERAD pathway: Leucine-zipper of ternary complex factor MIP1, Luminal-binding protein 4 (BiP), EGF domain-specific O-linked N-acetylglucosamine transferase, Probable E3 ubiquitin ligase SUD1, Vacuolar protein sorting-associated protein 28 homolog 2 and Calcineurin B-like protein 4. A model of BMYV-BiP interaction and a possible mechanism of partial resistance in the resistant genotype is illustrated in Fig. 8. MIP1 is a J-domain protein and it is a co-chaperone partner of the Hsp70 chaperone [67]. These proteins function in processes like protein folding, sorting, stabilization, translocation and refolding [68–70]. MIP1 proteins in *Nicotiana benthamiana* are reported to be associated with tobacco mosaic virus (TMV) movement protein (MP), resistance protein (Tm-2^2^) and resistance signaling component (SGT1) to assist in protein folding and maintaining protein structural stability. MIP1 is required for conferring resistance to TMV by functioning as co-chaperons as well as it is required for virus infection [71]. BiP is a key protein in the ERAD pathway where it identifies malformed proteins for repair or degradation [63]. Pathogens target the host proteins involved in the ER stress pathway in order to facilitate infection and BiPs are seen to regulate plant responses to both biotic and abiotic stresses [72–74]. A study done with PLRV revealed that the CP of the virus interacts with BiP [75]. A 7-amino-acid motif in the arginine-rich region of the CP N-terminus was identified to be docked within the substrate binding domain of BiP and this motif of CP was found to be conserved across viruses of 19 species of the genera *Luteovirus*, *Polerovirus* and *Enamovirus*, including BMYV. According to DeBlasio et al. [75], the CP-BiP interaction could reduce ER cytotoxicity generated by high level synthesis and accumulation of viral proteins. This may hold true for BMYV as well since it is also a polerovirus with a motif of CP interacting with the substrate binding domain of BiP and in the present study, there was a significant upregulation of the BiP gene in leaves of the resistant genotype at 21 DPI. In *N. benthamiana* plants infected with potato virus X (genus *Potexvirus*), overexpression of the BiP chaperone has been reported to suppress cell death induced by triple-gene-block protein 3 (TGBp3) [76, 77]. In our study, symptoms were not expressed in the resistant genotype, whereas in the susceptible genotype, we did see symptoms and death of the inoculated leaf by 28 DPI. BiP has been reported to be associated with the MP of bamboo mosaic virus (genus *Potexvirus*), and the findings revealed the role of BiP and calreticulin in the intracellular movement and spread of the virus in *N. benthamiana* [66].

Resistance proteins (R) sense the presence of pathogen effectors and they trigger signals leading to ETI by activation of hypersensitive response (HR) [78]. The resistant plants in this study differentially expressed R genes like EL10Ac3g07017 (Putative disease resistance protein RGA4) and EL10Ac3g07016 (Putative disease resistance protein RGA3), but no visible HR was observed in this genotype. It could indicate that this genotype might be conferring resistance to BMYV through the ETI pathway without the HR. In fact, plants are known to confer resistance against viruses through ETI pathway in the absence of HR [37]. EL10Ac5g11039 (Thioredoxin-like 1–2) was also seen to be significantly upregulated at multiple time points in the resistant genotype and downregulated in the susceptible genotype indicating a putative role in virus resistance. Thioredoxin enzymes have photoprotective mechanisms and they safeguard antioxidant enzymes from oxidative damage [79]. They are activated as the redox status of the cell undergoes change upon pathogen invasion. Infection by barley stripe mosaic virus (BSMV; genus *Hordeivirus*) triggers SA signalling mediated defence, upregulating the gene encoding *N. benthamiana* thioredoxin h-type1 (NbTRXh1) [80]. Overexpression of NbTRXh1 was found to impede BSMV infection and reduced level of NbTRXh1 resulted in higher levels of virus accumulation [80]. Cytochrome p450 (*CYP*) family genes were also seen to be uniquely upregulated in the resistant genotype. They have an important role in secondary metabolite production and plant defence to several biotic and abiotic stresses [81]. The CYP family gene *PAD3* is known to get activated in plants upon wounding by insects and in *A. thaliana*, *PAD3* regulates biosynthesis of camalexin, which confers resistance to the green peach aphid that transmits many viruses [82]. As a response to BMYV infection, 13 TF genes displayed unique differential expression in resistant plants, including genes of the bHLH, AP2-EREBP, MYB and NAC TF families. Apart from having other functions in plants, these TF families are reported to be involved in the transcriptional activity of virus-responsive genes, e.g., resistance (R) genes, genes linked to RNA silencing and also in translational suppression [83–89].

GO analysis revealed stress responsive biological pathways upregulated in old leaves at 21 DPI in the resistant genotype, where the top biological processes included the response to organic cyclic compound and response to SA. Organic cyclic compounds are secondary metabolites that plants normally produce in response to stress, which is also the case in virus-infected plants [90]. Salicylic acid is a critical hormone, being a signaling molecule, and it is known to be involved in plant immune responses and to have roles in activating defence genes, in DNA damage response and helping plants to mitigate the detrimental effects of virus infection [91]. Downregulated biological processes at 21 DPI in the old leaves of resistant plants exposed to the virus were mostly involved in pathways related to photosynthesis, but with very low gene count in comparison to the susceptible genotype. The explanation could be that the plant is shutting down photosynthesis to limit virus multiplication, an indirect effect of infection or because the virus disturbs processes in the chloroplast [92]. Also, downregulation of genes related to photosynthesis and chloroplasts in parallel with the upregulation of defence response genes (genes for synthesis pathways of JA, SA and ethylene) is commonly seen in infections by various pathogens, suggesting that reduction in photosynthesis is an adaptive response to biotic stress [93].

One of the most common virus symptoms seen in plants is that of chlorosis and it is often associated with changes in chloroplast structures, damage to photosystems І and II and reduction in chlorophyll content [94, 95]. Response to light intensity and photosynthesis-related pathways were observed among the top downregulated biological processes at 14 DPI in the susceptible genotype. In addition, photosynthesis was found to be the KEGG pathway with highest enrichment in old leaves of susceptible plants. This was expected because leaf chlorosis is a symptom of the disease and it is often associated with disruption of chloroplast- and photosynthesis-related pathways in plants [96]. The progression of symptoms along with downregulation of genes related to chloroplast and photosynthesis suggests that there may be weaker host defence responses to BMYV in susceptible plants [97]. A similar transcriptional response underlying symptom expression has been seen in susceptible soybean plants infected with soybean mosaic virus and in tobacco plants infected with cucumber mosaic virus [98, 99]. In addition, in the susceptible genotype, the linked GO IDs in the interactive graph generated by the REVIGO web tool were mostly related to stress damage response and top interacting biological processes included DNA damage response, DNA templated DNA replication, DNA metabolic process and DNA replication. DNA metabolic process was the top biological process of old leaves at 21 DPI in the susceptible genotype and DNA replication was one among the significantly enriched KEGG pathways. Plant immune responses to several biotic stresses and the DNA damage response are connected in numerous ways [100, 101]. Virus infection could lead to production of reactive oxygen species (ROS) causing oxidative DNA damage and accumulated pressure on the host’s cellular machinery resulting in DNA replication stress and damage. Genes encoding chalcone synthase were found to be significantly upregulated in old leaves of resistant plants at 21 DPI and this enzyme belongs to the flavonoid/isoflavanoid biosynthesis pathway. Interestingly, the KEGG pathway significantly enriched in the susceptible genotype also included that of biosynthesis of secondary metabolites. This shows that genes involved in the production of secondary metabolites could undergo significant expression changes in both genotypes indicating that they play a role in the response to BMYV infection. The accumulation of these secondary metabolites and their role in conferring resistance to pathogens and pests in different plants, including sugar beet, are well studied and established [102, 103].

## Conclusions

In this study, we analysed two beet genotypes and delved into the transcriptomic changes in response to BMYV infection at several time points after inoculation in inoculated and young leaves. In the susceptible genotype, BMYV infection resulted in symptoms, high virus titre and many DEGs, while in the resistant genotype, there were no symptoms, the virus titre was low, and there were fewer DEGs, which indicates partial resistance. The virus quantification results together with the DEGs identified in the resistant genotype at different time points and leaf ages shed light on the possible molecular mechanisms operating towards partial resistance against BMYV. The unique differential expression of TF genes may be a crucial factor for the observed different responses to BMYV infection in susceptible and resistant plants. Some of the genes significantly upregulated in the resistant genotype encode proteins involved in protein processing in the ER and this could be one mechanism contributing to the reduced virus level. Gene ontology analysis and KEGG pathways revealed clear differences between genotypes in the biological pathways in response to virus infection. The resistant genotype showed more of the stress-responsive biological pathways related to plant immunity whereas in the susceptible genotype it was more of the processes involved in DNA damage response and photosynthesis. Transient overexpression of selected candidate genes or knock-out studies using model species could further help in proving their functions predicted with the transcriptomic analysis.

## Supplementary Information

Additional file 1. (A) Experimental set up illustrating the four treatments of beet seedlings: aphids with virus (Inoculated), aphids without virus (Healthy), plants without exposure to aphids or virus (Non-inoculated) and plants treated with only insecticide (Insecticide control). The different colours indicate the separate treatments and the arrows indicate the direction followed during aphid exposure within each treatment. (B-D) Arrangement of plants in different trays for sample collection at various time points with the use of fleece cover to avoid cross-contamination.

Additional file 2. Transcriptome statistics of sequencing reads of RNA from beet leaves in study of response to BMYV infection. The average number of reads in millions processed and mapped to the EL10 reference genome of sugar beet is shown on the left X axis with average of three biological replicates for all treatments, time points and both genotypes. The X axis on the right side shows the average percentage of mapped reads. The Y axis shows the sample names: S, susceptible; R, resistant; O, old leaf; Y, young leaf; D, number of days; I, inoculated; H, healthy; ins, insecticide. Red and green dots indicate susceptible and resistant genotypes, respectively.

Additional file 3. Principal component analysis (PCA) for the transcriptomic data of old (A) and young (B) leaves from both genotypes (distinguished by mark sizes) in response to BMYV infection. The PCA includes all time points (shapes) and treatments (colours).

Additional file 4. Biological processes upregulated at 21 DPI in old leaves of the susceptible genotype in response to BMYV infection (P adj value < 0.05).

Additional file 5. KEGG pathways enriched in the susceptible genotype in response to BMYV infection combining all the time points as well as old and young leaves.

Additional file 6. Interactive representative GO IDs obtained using the REVIGO web tool for resistant (A) and susceptible (B) genotype, in response to BMYV infection combining all the time points as well as old and young leaves.

Additional file 7. Leaf samples used for RNAseq analysis.

Additional file 8. Differentially expressed genes (DEGs; FDR<0.05 and log2FoldChange >1 or <-1) upregulated in the resistant genotype and downregulated in the susceptible genotype as well as the DEGs in response to BMYV infection that are unique to the resistant genotype.

Additional file 9. Differentially expressed genes (FDR<0.05 and log2FoldChange >1 or < -1) in the susceptible genotype with the largest change in response to BMYV infection.

Additional file 10. Differentially expressed genes (FDR<0.05 and log2FoldChange >1 or < -1) in the resistant genotype in response to BMYV infection.

Additional file 11. Differentially expressed genes encoding transcription factors in the resistant genotype in response to BMYV infection.

Additional file 12. Biological processes, cellular components and molecular functions up- or downregulated in the resistant genotype in response to BMYV infection.

Additional file 13. Biological processes, cellular components and molecular functions up- or downregulated in the susceptible genotype in response to BMYV infection.

## Data Availability

Raw sequencing data can be obtained from National Centre for Biotechnological Information (NCBI) under the Bioproject accession number PRJEB80223. All other data are available upon request.

## Abbreviations

BChV: Beet chlorosis virus
BiP: Luminal binding protein
BMYV: Beet mild yellowing virus
BWYV: Beet western yellows virus
BYV: Beet yellows virus
CP: Coat protein
DEGs: Differentially expressed genes
DPI: Days post-inoculation
ELISA: Enzyme-linked immunosorbent assay
ER: Endoplasmic reticulum
ERAD: Endoplasmic reticulum associated degradation
ETI: Effector triggered immunity
FDR: False discovery rate
GO: Gene ontology
JA: Jasmonic acid
KEGG: Kyoto Encyclopedia of genes and genomes
PCA: Principle component analysis
RIN: RNA integrity number
RNAseq: RNA sequencing
SA: Salicylic acid
UPS: Ubiquitin/26S proteasome system
Vsps: Vacuolar sorting associated proteins
VY: Virus yellows
