# Supplementary figures and images for: Transcriptomic responses of beet to infection by beet mild yellowing virus
Source: BMC Plant Biol. 2025 Oct 21;25:1406. doi: 10.1186/s12870-025-07514-6 (PMC12538817; doi:10.1186/s12870-025-07514-6)

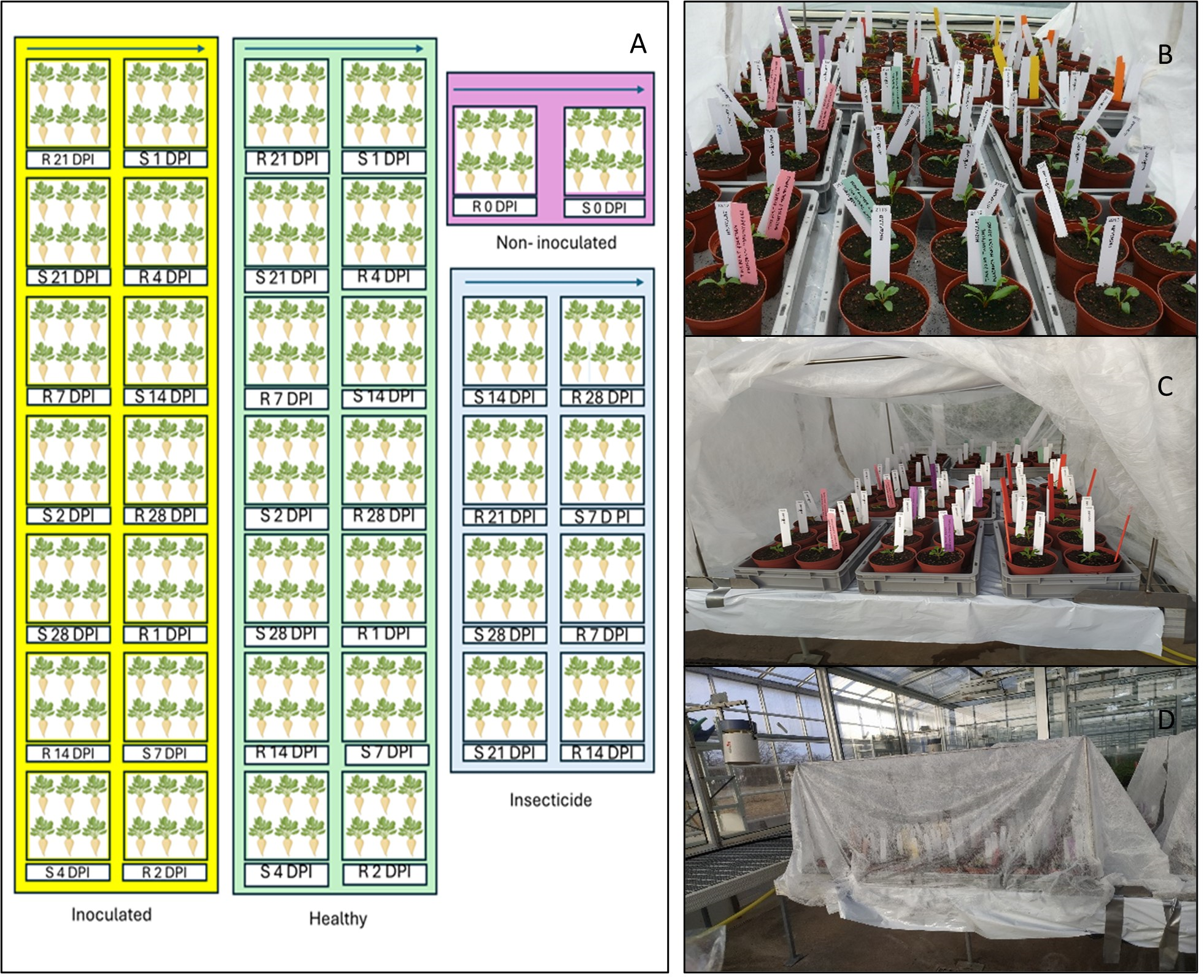

Supplement: Supplementary file 1 — Additional file 1. (A) Experimental set up illustrating the four treatments of beet seedlings: aphids with virus (Inoculated), aphids without virus (Healthy), plants without exposure to aphids or virus (Non-inoculated) and plants treated with only insecticide (Insecticide control). The different colours indicate the separate treatments and the arrows indicate the direction followed during aphid exposure within each treatment. (B-D) Arrangement of plants in different trays for sample collection at various time points with the use of fleece cover to avoid cross-contamination. [file 12870_2025_7514_MOESM1_ESM.tif]

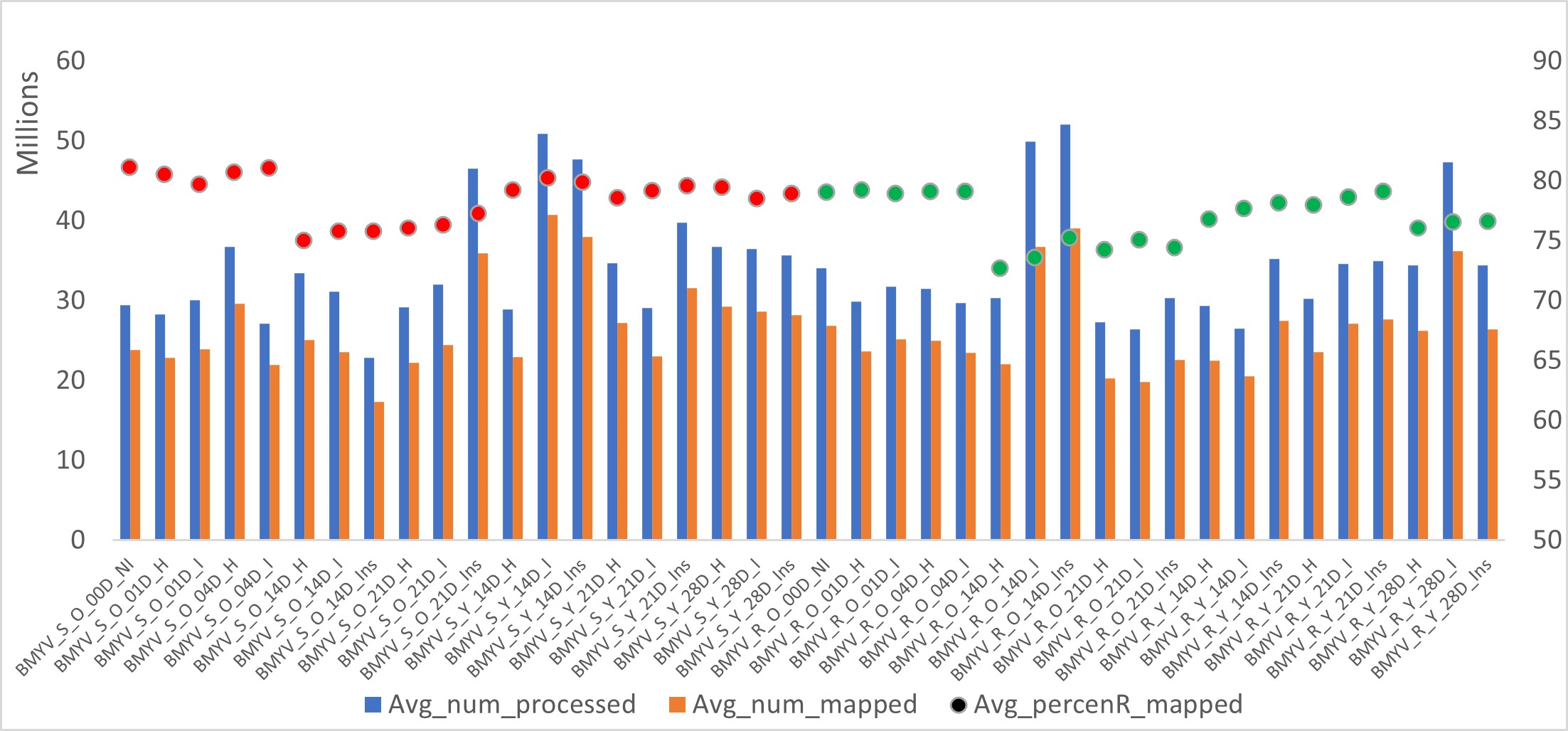

Supplement: Supplementary file 2 — Additional file 2. Transcriptome statistics of sequencing reads of RNA from beet leaves in study of response to BMYV infection. The average number of reads in millions processed and mapped to the EL10 reference genome of sugar beet is shown on the left X axis with average of three biological replicates for all treatments, time points and both genotypes. The X axis on the right side shows the average percentage of mapped reads. The Y axis shows the sample names: S, susceptible; R, resistant; O, old leaf; Y, young leaf; D, number of days; I, inoculated; H, healthy; ins, insecticide. Red and green dots indicate susceptible and resistant genotypes, respectively. [file 12870_2025_7514_MOESM2_ESM.tif]

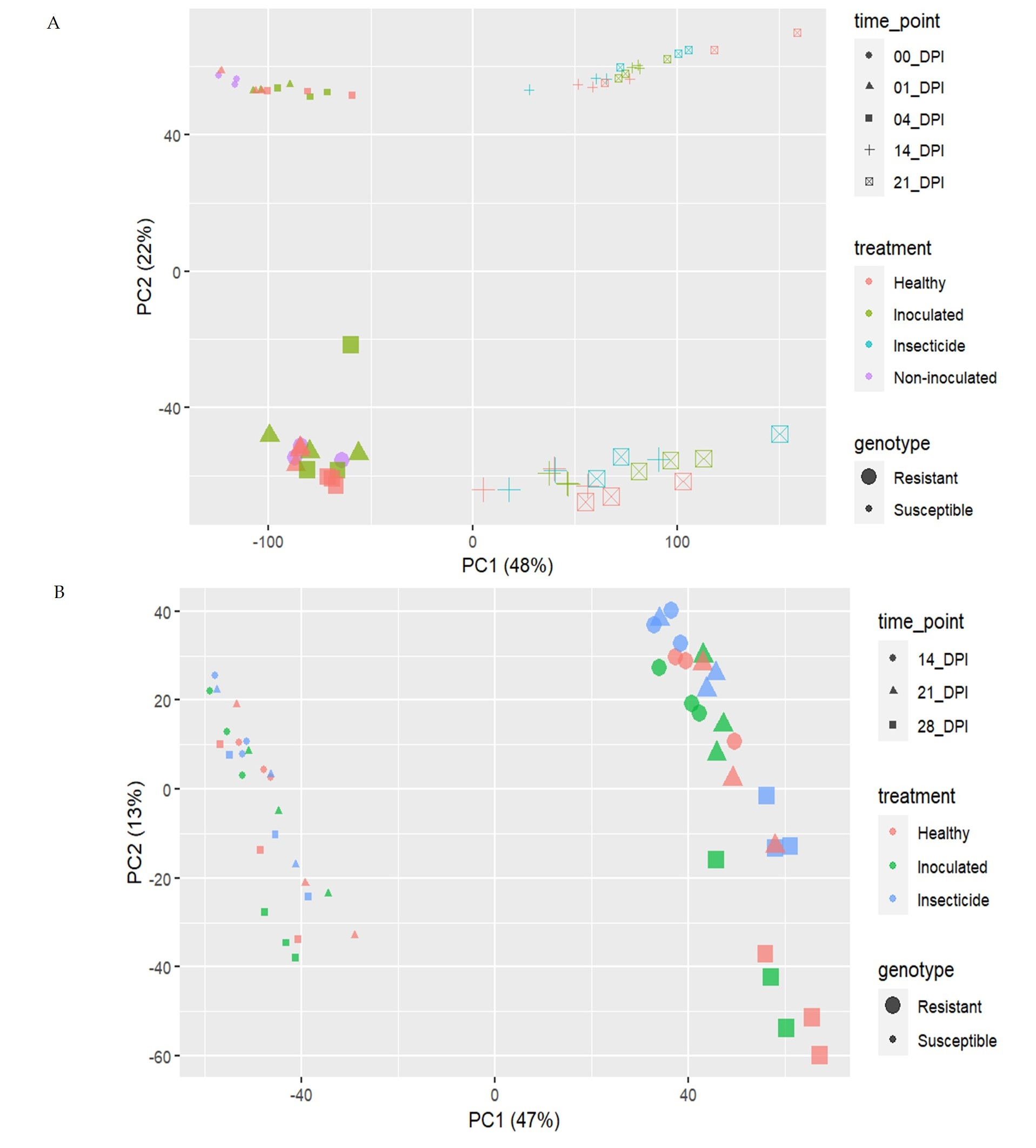

Supplement: Supplementary file 3 — Additional file 3. Principal component analysis (PCA) for the transcriptomic data of old (A) and young (B) leaves from both genotypes (distinguished by mark sizes) in response to BMYV infection. The PCA includes all time points (shapes) and treatments (colours). [file 12870_2025_7514_MOESM3_ESM.tif]

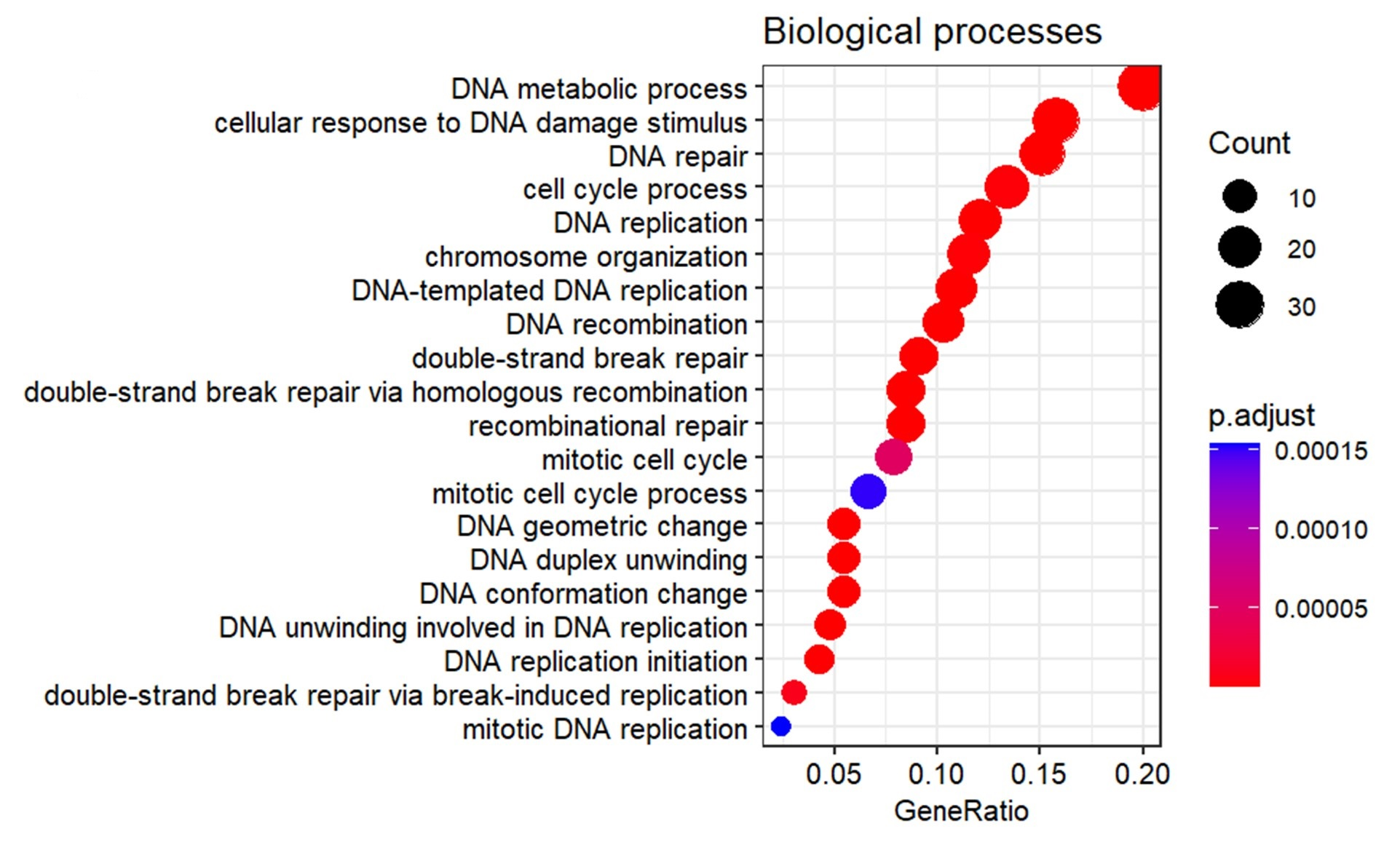

Supplement: Supplementary file 4 — Additional file 4. Biological processes upregulated at 21 DPI in old leaves of the susceptible genotype in response to BMYV infection (P adj value < 0.05). [file 12870_2025_7514_MOESM4_ESM.tif]

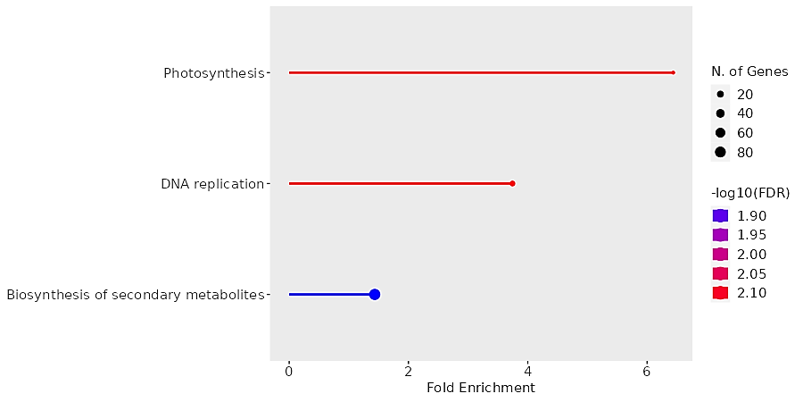

Supplement: Supplementary file 5 — Additional file 5. KEGG pathways enriched in the susceptible genotype in response to BMYV infection combining all the time points as well as old and young leaves. [file 12870_2025_7514_MOESM5_ESM.tif]

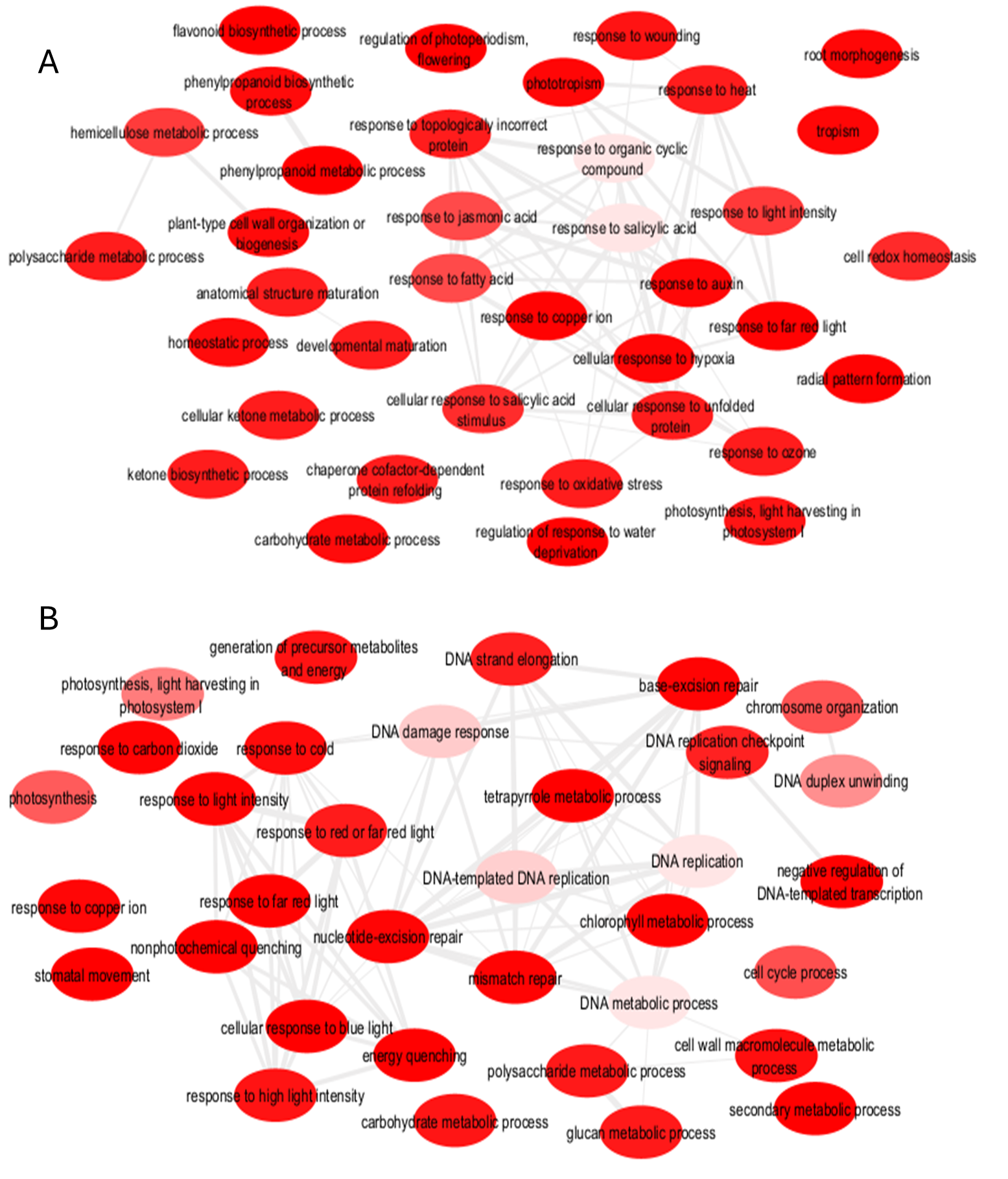

Supplement: Supplementary file 6 — Additional file 6. Interactive representative GO IDs obtained using the REVIGO web tool for resistant (A) and susceptible (B) genotype, in response to BMYV infection combining all the time points as well as old and young leaves. [file 12870_2025_7514_MOESM6_ESM.tif]
