# Supplementary material for: Transcriptomic responses of beet to infection by beet mild yellowing virus
Source: BMC Plant Biol. 2025 Oct 21;25:1406. doi: 10.1186/s12870-025-07514-6 (PMC12538817; doi:10.1186/s12870-025-07514-6)
Supplement: Supplementary file 7 — Additional file 7. Leaf samples used for RNAseq analysis. [file 12870_2025_7514_MOESM7_ESM.docx]

Additional file 7. Leaf samples used for RNAseq analysis

| Genotype | DPI | Treatment (Code)^a^ | | | |
| --- | --- | --- | --- | --- | --- |
|  |  | Non-inoculated | Inoculated | Healthy | Insecticide |
| Resistant | 0 | BMYV_R_O_00D_NI |  |  |  |
|  | 1 |  | BMYV_R_O_01D_I | BMYV_R_O_01D_H |  |
|  | 4 |  | BMYV_R_O_04D_I | BMYV_R_O_04D_H |  |
|  | 14 |  | BMYV_R_O_14D_I | BMYV_R_O_14D_H | BMYV_R_O_14D_Ins |
|  |  |  | BMYV_R_Y_14D_I | BMYV_R_Y_14D_H | BMYV_R_Y_14D_Ins |
|  | 21 |  | BMYV_R_O_21D_I | BMYV_R_O_21D_H | BMYV_R_O_21D_Ins |
|  |  |  | BMYV_R_Y_21D_I | BMYV_R_Y_21D_H | BMYV_R_Y_21D_Ins |
|  | 28 |  | BMYV_R_Y_28D_I | BMYV_R_Y_28D_H | BMYV_R_Y_28D_Ins |
| Susceptible | 0 | BMYV_S_O_00D_NI |  |  |  |
|  | 1 |  | BMYV_S_O_01D_I | BMYV_S_O_01D_H |  |
|  | 4 |  | BMYV_S_O_04D_I | BMYV_S_O_04D_H |  |
|  | 14 |  | BMYV_S_O_14D_I | BMYV_S_O_14D_H | BMYV_S_O_14D_Ins |
|  |  |  | BMYV_S_Y_14D_I | BMYV_S_Y_14D_H | BMYV_S_Y_14D_Ins |
|  | 21 |  | BMYV_S_O_21D_I | BMYV_S_O_21D_H | BMYV_S_Y_21D_Ins |
|  |  |  | BMYV_S_Y_21D_I | BMYV_S_Y_21D_H | BMYV_S_O_21D_Ins |
|  | 28 |  | BMYV_S_Y_28D_I | BMYV_S_Y_28D_H | BMYV_S_Y_28D_Ins |
|  |  |  |  |  |  |

^a^The code follows the pattern of the virus name, followed by genotype, leaf age, time point and treatment. O indicates

old (inoculated) leaves and Y indicates young (systemic) leaves, respectively
