# Supplementary material for: Transcriptomic responses of beet to infection by beet mild yellowing virus
Source: BMC Plant Biol. 2025 Oct 21;25:1406. doi: 10.1186/s12870-025-07514-6 (PMC12538817; doi:10.1186/s12870-025-07514-6)
Supplement: Supplementary file 8 — Additional file 8. Differentially expressed genes (DEGs; FDR<0.05 and log2FoldChange >1 or <-1) upregulated in the resistant genotype and downregulated in the susceptible genotype as well as the DEGs in response to BMYV infection that are unique to the resistant genotype. [file 12870_2025_7514_MOESM8_ESM.docx]

Additional file 8. Differentially expressed genes (DEGs; FDR<0.05 and log2FoldChange > 1 or < -1) upregulated in the resistant genotype and downregulated in the susceptible genotype as well as the DEGs unique to the resistant genotype

| **Names** | **Total** | **EL10 gene IDs** | **Sugar beet annotation** | **Arabidopsis IDs, annotation and gene symbols** |
| --- | --- | --- | --- | --- |
| S Down R up | 5 | EL10Ac8g18366 | hypothetical protein | AT4G26288.1, Oxidative stress 3 |
|  |  | EL10Ac8g19221 | Putative beta-galactosidase | AT4G26140.1, BGAL12, beta-galactosidase 12 |
|  |  | EL10Ac5g11039 | Thioredoxin-like 1-2, chloroplastic | AT5G61440.1, ACHT5, atypical CYS HIS rich thioredoxin 5 |
|  |  | EL10Ac5g12806 |  | AT3G61460.1, BRH1, brassinosteroid-responsive RING-H2 |
|  |  | EL10Ac8g20350 | Stem-specific protein TSJT1 | AT4G27450.1, Aluminium induced protein with YGL and LRDR motifs |
| R Up | 43 | EL10Ac3g07016 | Putative disease resistance protein RGA3 | AT1G53350.1, Disease resistance protein (CC-NBS-LRRclass) family |
|  |  | EL10Ac8g18447 |  | AT4G20050.2, QRT3, Pectinlyase-like super family protein |
|  |  | EL10Ac4g09150 | Auxin-responsive protein IAA4 | AT5G43700.1, ATAUX2-11, IAA4, AUX/IAA transcriptional regulator family protein |
|  |  | EL10Ac2g03638 | Cytochrome P450 CYP73A100 | AT2G30490.1, ATC4H, C4H, CYP73A5, REF3, cinnamate-4-hydroxylase |
|  |  | EL10Ac2g04338 | Cytokinin riboside 5'-monophosphate phosphoribohydrolase LOG8 | AT5G11950.2, Putative lysine decarboxylase family protein |
|  |  | EL10Ac8g18716 | hypothetical protein | AT3G43955.1, Transposable element gene |
|  |  | EL10Ac5g10542 | hypothetical protein | AT5G11460.1, Protein of unknown function (DUF581) |
|  |  | EL10Ac6g15151 | Leucine-zipper of ternary complex factor MIP1 | AT4G37080.3, Protein of unknown function, (DUF547) |
|  |  | EL10Ac3g07108 | EGF domain-specific O-linked N-acetylglucosamine transferase | AT2G41640.1, Glycosyl transferase family 61 protein |
|  |  | EL10Ac4g07519 | Probable LRR receptor-like serine/threonine-protein kinase At4g26540 | AT5G56040.2, Leucine-rich receptor-like protein kinase family protein |
|  |  | EL10Ac8g18590 | Cytochrome P450 734A1 | AT2G26710.1, BAS1, CYP734A1, CYP72B1, Cytochrome P450 super family protein |
|  |  | EL10Ac1g00851 | Chalcone synthase | AT5G13930.1, CHS, TT4, ATCHS, Chalcone and stilbene synthase family protein |
|  |  | EL10Ac3g06084 | Probable E3 ubiquitin ligase SUD1 | AT5G38070.1, RING/FYVE/PHDzinc finger super family protein |
|  |  | EL10Ac8g18621 | Dynein light chain, cytoplasmic | AT5G20110.1, Dynein light chain type 1 family protein |
|  |  | EL10Ac1g00725 | Protein of unknown function (DUF581) | AT5G47060.1, Protein of unknown function (DUF581) |
|  |  | EL10Ac3g07015 | Auxin efflux carrier component 3 | AT1G70940.1, PIN3, ATPIN3, Auxin efflux carrier family protein |
|  |  | EL10Ac8g20617 | Vacuolar protein sorting-associated protein 28 homolog 2 | AT4G21560.3, VPS28-1,vacuolar protein sorting-associated protein28 homolog1 |
|  |  | EL10As5g23509 |  | AT1G67730.1, YBR159, KCR1, ATKCR1, beta-keto acylreductase1 |
|  |  | EL10Ac6g13175 | Homeobox-leucine zipper protein ATHB-13 | AT1G26960.1, AtHB23, HB23, homeobox protein 23 |
|  |  | EL10Ac9g22303 | Root cap | AT3G19430.1, late embryogenesis abundant protein-related/LEAprotein-related |
|  |  | EL10As7g23811 |  | AT4G02100.1, Heat shock protein DnaJ with tetratricopeptide repeat |
|  |  | EL10Ac5g12002 | Probable xyloglucan endotransglucosylase/hydrolase protein 30 | AT1G32170.1, XTR4, XTH30, xyloglucan endotransglucosylase/hydrolase30 |
|  |  | EL10Ac6g13210 | Ribonuclease 1 | AT2G02990.1, RNS1, ATRNS1, ribonuclease1 |
|  |  | EL10As19g24172 |  | AT3G51050.1, FG-GAP repeat-containing protein |
|  |  | EL10Ac2g04662 | UDP-glycosyltransferase 76C2 | AT3G55710.1, UDP-Glycosyl transferase super family protein |
|  |  | EL10Ac6g13275 | Protein ENHANCED DISEASE RESISTANCE 2-like | AT5G39430.1, Protein of unknown function (DUF1336) |
|  |  | EL10Ac8g19013 | Auxin response factor 19 | AT5G20730.2, NPH4, MSG1, IAA21, ARF7, TIR5, BIP, Transcriptional factor B3 family protein/auxin-responsive factorAUX/IAA-related |
|  |  | EL10Ac7g16456 | Dormancy/auxin associated protein | AT3G26840.1, Esterase/lipase/thioesterase family protein |
|  |  | EL10Ac8g18493 |  | AT5G19650.1, ATOFP8, OFP8, ovate family protein 8 |
|  |  | EL10Ac3g05144 |  | AT1G07460.1, ConcanavalinA-like lectin family protein |
|  |  | EL10Ac3g07017 | Putative disease resistance protein RGA4 | AT5G43730.1, Disease resistance protein (CC-NBS-LRR class) family |
|  |  | EL10Ac6g13345 | Beta-glucosidase 12 | AT3G18070.2, BGLU43, beta glucosidase 43 |
|  |  | EL10Ac5g10982 | Transcription factor TCP15 | AT1G58100.1, TCP family transcription factor |
|  |  | EL10Ac3g05286 | Calcineurin B-like protein 4 | AT5G24270.2, SOS3, Calcium-binding EF-hand family protein |
|  |  | EL10Ac4g09930 | Luminal-binding protein 4 | AT1G09080.2, BIP3, Heat shock protein 70 (Hsp70) family protein |
|  |  | EL10Ac3g07413 |  | AT5G42680.2, Protein of unknown function, DUF617 |
|  |  | EL10Ac4g08054 |  | AT1G24625.1, ZFP7, zinc finger protein 7 |
|  |  | EL10Ac9g22167 | Protein of unknown function (DUF1005) | AT1G10020.1, Protein of unknown function (DUF1005) |
|  |  | EL10Ac7g15822 |  | AT1G76930.2, ATEXT4, ORG5, ATEXT1, EXT1, EXT4, extensin 4 |
