# Supplementary material for: Transcriptomic responses of beet to infection by beet mild yellowing virus
Source: BMC Plant Biol. 2025 Oct 21;25:1406. doi: 10.1186/s12870-025-07514-6 (PMC12538817; doi:10.1186/s12870-025-07514-6)
Supplement: Supplementary file 9 — Additional file 9. Differentially expressed genes (FDR<0.05 and log2FoldChange >1 or < -1) in the susceptible genotype with the largest change in response to BMYV infection. [file 12870_2025_7514_MOESM9_ESM.docx]

Additional file 9. Differentially expressed genes (FDR<0.05 and log2FoldChange > 1 or < -1) in the susceptible genotype with the largest change in response to BMYV infection

| \| **Gene ID** \| \| --- \| | **Log2foldchange** | **Time point (DPI)** | **Sugar beet annotation** |
| --- | --- | --- | --- | --- |
| \| EL10Ac3g05021 \| \| --- \| \| EL10Ac5g10885 \| \| EL10Ac8g18462 \| \| EL10Ac4g09226 \| \| EL10Ac6g14542 \| \| EL10Ac7g18118 \| \| EL10Ac9g22129 \| \| EL10Ac3g06705 \| \| EL10Ac2g03809 \| \| EL10Ac6g14312   \| EL10Ac9g21200 \| \| --- \| \| EL10Ac6g15528 \| \| EL10Ac4g10207 \| \| EL10Ac9g21714 \| \| EL10Ac1g02186 \| \| EL10Ac4g10066 \| \| EL10Ac6g14649 \| \| EL10Ac8g20495 \| \| EL10Ac6g15503 \| \| EL10Ac9g21985 \| \|  \| \| | \| 4.1366 \| \| --- \| \| 3.6222 \| \| 3.5657 \| \| 3.1781 \| \| 3.0182 \| \| 3.0152 \| \| 2.9836 \| \| 2.9459 \| \| 2.8779 \| \| 2.8426 \| \| -4.0056 \| \| \| -3.3235 \| \| \| -3.2477 \| \| \| -2.8017 \| \| \| -2.7595 \| \| \| -2.7415 \| \| \| -2.6405 \| \| \| -2.6099 \| \| \| -2.5487 \| \| \| -2.5042 \| \| | \| 21 \| \| \| --- \| --- \| \| 21 \| \| \| 21 \| \| \| 21 \| \| \| 14 \| \| \| 21 \| \| \| 14 \| \| \| 14 \| \| \| 14 \| \| \| 21 \| \| \| 14 \| \| 21 \| \| 4 \| \| 14 \| \| 14 \| \| 21 \| \| 21 \| \| 21 \| \| 21 \| \| 14 \| | \| Uncharacterized protein L728 \| \| \| --- \| --- \| \| Defensin-like protein \| \| \| MLP-like protein 43 \| \| \| Protein of unknown function (DUF2775) \| \| \|  \| \| \| Beta-amyrin synthase \| \| \| Cysteine-rich TM module stress tolerance \| \| \| Trypsin inhibitor A \| \| \|  \| \| \| Reticulon-like protein B9 \| \| \| hypothetical protein \| \| Mannose/glucose-specific lectin \| \| Glucan endo-1,3-beta-glucosidase \| \| Transcription factor RADIALIS \| \|  \| \| Jasmonate-induced protein homolog \| \| Jasmonate-induced protein homolog \| \| hypothetical protein \| \| Mannose/glucose-specific lectin \| |
