# Supplementary material for: Transcriptomic responses of beet to infection by beet mild yellowing virus
Source: BMC Plant Biol. 2025 Oct 21;25:1406. doi: 10.1186/s12870-025-07514-6 (PMC12538817; doi:10.1186/s12870-025-07514-6)
Supplement: Supplementary file 10 — Additional file 10. Differentially expressed genes (FDR<0.05 and log2FoldChange >1 or < -1) in the resistant genotype in response to BMYV infection. [file 12870_2025_7514_MOESM10_ESM.docx]

Additional file 10. Differentially expressed genes (FDR<0.05 and log2FoldChange > 1 or < -1) in the resistant genotype in response to BMYV infection

| **EL10ID** | **Log2Fold**  **Change** | **Chr** | **Annotation** | **Arabidopsis ID and gene symbols** |
| --- | --- | --- | --- | --- |
| **4 DPI OLD** |  |  |  |  |
| EL10Ac2g04733 | 1.0112 | 2 | Expansin-A4 | AT2G28950.1, ATEXPA6, ATEXP6, ATHEXPALPHA1.8, EXPA6, expansinA6 |
| EL10Ac6g14883 | -1.0097 | 6 | Probable vacuolar amino acid transporter YPQ3 | AT4G36850.1, PQ-loop repeat family protein/transmembrane family protein |
| EL10Ac1g01063 | -1.0647 | 1 | Basic endochitinase | AT3G12500.1, ATHCHIB, PR3, PR-3, CHI-B, B-CHI, HCHIB, basicchitinase |
| EL10Ac1g02186 | -1.0685 |  |  | AT2G17880.1, Chaperone DnaJ-domain super family protein |
| **14 DPI OLD** |  |  |  |  |
| EL10Ac7g15822 | 1.3234 |  |  | AT1G76930.2, ATEXT4, ORG5, ATEXT1, EXT1, EXT4, extensin4 |
| EL10Ac8g20350 | 1.1466 | 8 | Stem-specific protein TSJT1 | AT4G27450.1, Aluminium induced protein with YGL and LRDR motifs |
| EL10Ac8g19221 | 1.1081 | 8 | Putative beta-galactosidase | AT4G26140.1, BGAL12, beta-galactosidase12 |
| EL10Ac2g04345 | 1.0863 | 2 | Cytochrome b5 domain-containing protein RLF {ECO:0000305} | AT3G08030.2, Protein of unknown function, DUF642 |
| EL10Ac4g09980 | -1.0204 | 4 | Putative auxin efflux carrier component 8 | AT5G16530.1, PIN5, Auxin efflux carrier family protein |
| EL10Ac4g08503 | -1.0529 |  |  | AT1G30100.1, NCED5, ATNCED5, nine-cis-epoxy carotenoid dioxygenase5 |
| **21 DPI OLD** |  |  |  |  |
| EL10Ac2g03638 | 1.2717 | 2 | Cytochrome P450 CYP73A100 | AT2G30490.1, ATC4H, C4H, CYP73A5, REF3, cinnamate-4-hydroxylase |
| EL10Ac1g00851 | 1.2432 | 1 | Chalcone synthase | AT5G13930.1, CHS, TT4, ATCHS, Chalcone and stilbene synthase family protein |
| EL10Ac6g13427 | 1.2081 | 6 | Glyceraldehyde-3-phosphate dehydrogenase, cytosolic | AT3G04120.1, GAPC, GAPC-1, GAPC1, glyceraldehyde-3-phosphate dehydrogenase C subunit 1 |
| EL10Ac7g15992 | 1.1750 | 7 | Heat shock cognate 70 kDa protein 2 | AT3G12580.1, HSP70, ATHSP70, heat shock protein 70 |
| EL10Ac8g18447 | 1.1377 | 8 | Polygalacturonase QRT3 | AT4G20050.2, QRT3, Pectinlyase-like super family protein |
| EL10Ac6g15151 | 1.1031 | 6 | Leucine-zipper of ternary complex factor MIP1 | AT4G37080.3, Protein of unknown function, DUF547 |
| EL10Ac7g16802 | 1.0880 |  |  | AT5G09750.1, HEC3, basichelix-loop-helix (bHLH) DNA-binding super family protein |
| EL10Ac6g13101 | 1.0864 |  |  | AT4G27560.1, UDP-Glycosyl transferase super family protein |
| EL10Ac6g13275 | 1.0708 | 6 | Protein ENHANCED DISEASE RESISTANCE 2-like | AT5G39430.1, Protein of unknown function (DUF1336) |
| EL10Ac6g13345 | 1.0657 | 6 | Beta-glucosidase 12 | AT3G18070.2, BGLU43, betaglucosidase 43 |
| EL10Ac4g09930 | 1.0595 | 4 | Luminal-binding protein 4 | AT1G09080.2, BIP3, Heat shock protein 70 (Hsp70) family protein |
| EL10Ac3g07108 | 1.0549 | 3 | EGF domain-specific O-linked N-acetylglucosamine transferase | AT2G41640.1, Glycosyl transferase family 61 protein, |
| EL10Ac4g08825 | 1.0347 | 4 | Sieve element occlusion N-terminus | AT3G01680.1 |
| EL10Ac5g11039 | 1.0236 | 5 | Thioredoxin-like 1-2, chloroplastic | AT5G61440.1, ACHT5, atypical CYS HIS rich thioredoxin 5 |
| EL10Ac7g16456 | 1.0130 | 7 | Dormancy/auxin associated protein | AT3G26840.1, Esterase/lipase/thioesterase family protein |
| EL10Ac9g21951 | 1.0038 | 9 | Acyl-protein thioesterase 2 | AT3G15650.1, alpha/beta-Hydrolases super family protein |
| EL10Ac3g05369 | -1.0027 | 3 | Acidic endochitinase | AT5G24090.1, ATCHIA, CHIA, chitinase A |
| EL10Ac3g06110 | -1.0045 | 3 | hypothetical protein | AT3G45610.1, Dof-type zinc finger DNA-binding family protein |
| EL10Ac4g09067 | -1.0052 | 4 | Activating signal cointegrator 1 | AT2G20410.1, RNA-binding ASCH domain protein |
| EL10Ac3g05449 | -1.0152 | 3 | BAHD acyltransferase DCR | AT5G23940.1, EMB3009, PEL3, DCR, HXXXD-typeacyl-transferase family protein |
| EL10Ac2g03544 | -1.0196 | 2 | Ethylene-responsive transcription factor RAP2-7 | AT2G28550.2, RAP2.7, TOE1, related to AP2.7 |
| EL10Ac9g22710 | -1.0251 | 9 | Chlorophyll a-b binding protein 36, chloroplastic | AT2G05100.1, LHCB2.1, LHCB2, photosystem II light harvesting complex gene2.1 |
| EL10Ac7g18156 | -1.0316 |  |  | AT1G51080.1, LOCATED IN:chloroplast |
| EL10Ac3g06844 | -1.0338 | 3 | Putative 4-hydroxy-4-methyl-2-oxoglutarate aldolase 3 | AT5G56260.1, Ribonuclease E inhibitor RraA/Dimethyl menaquinone methyl transferase |
| EL10Ac4g08911 | -1.0397 | 4 | Butyrate--CoA ligase AAE11, peroxisomal | AT1G65880.1, BZO1, benzoyl oxyglucosinolate 1 |
| EL10Ac4g08118 | -1.0408 | 4 | Proto chlorophyllide reductase, chloroplastic | AT5G54190.2, PORA, protochlorophyll ideoxidoreductase A |
| EL10Ac5g12318 | -1.0487 | 5 | Nudix hydrolase 8 | AT5G47240.1, atnudt8, NUDT8, nudix hydrolase homolog 8 |
| EL10Ac3g05180 | -1.0536 | 3 | Ribosome-binding factor PSRP1, chloroplastic | AT5G24490.1, 30S ribosomal protein, putative |
| EL10Ac8g19822 | -1.0828 | 8 | Subtilisin-like protease SBT5.3 | AT5G45650.1, subtilase family protein |
| EL10Ac8g18899 | -1.1192 | 8 | Putative glutamine amido transferase YLR126C | AT2G23970.1, Class I glutamine amido transferase-like super family protein |
| EL10Ac5g11259 | -1.1320 | 5 | Gastric triacylglycerol lipase | AT1G73920.2, alpha/beta-Hydrolases super family protein |
| EL10As6g23687 | -1.1438 |  |  | AT5G28845.1, transposable element gene |
| EL10Ac9g21501 | -1.1522 |  |  | AT1G22370.2, AtUGT85A5, UGT85A5, UDP-glucosyl transferase 85 A5 |
| EL10Ac8g19352 | -1.1623 | 8 | Chlorophyll a-b binding protein CP24, chloroplastic | AT1G15820.1, LHCB6, CP24, light harvesting complex photosystemII subunit 6 |
| EL10Ac3g05746 | -1.1896 |  |  | AT5G49680.2, Golgi-body localisation protein domain; RNA polII promoter Fmp27 protein domain |
| EL10Ac8g19835 | -1.2025 | 8 | GDSL esterase/lipase At5g45670 | AT5G45670.1, GDSL-like Lipase/Acyl hydrolase super family protein |
| EL10Ac8g20539 | -1.2033 | 8 | Jacalin-like lectin domain | AT5G12130.1, PDE149, ATTERC, integral membrane Ter C family protein |
| EL10Ac7g16484 | -1.2218 | 7 | Protein of unknown function (DUF581) | AT1G53903.1, Protein of unknown function (DUF581) |
| EL10Ac9g21921 | -1.2669 | 9 | Glyoxalase-like domain | AT1G80160.2, Lactoyl glutathione lyase/glyoxalaseI family protein |
| EL10Ac2g03503 | -1.3239 | 2 | Vinorine synthase | AT4G15390.1, HXXXD-typeacyl-transferase family protein |
| EL10Ac8g19657 | -1.3573 |  |  | AT1G30135.1, JAZ8, TIFY5A, jasmonate-zim-domainprotein 8 |
| EL10Ac2g03669 | -1.4245 | 2 | Endonuclease V | AT4G31150.2, endonuclease V family protein |
| EL10Ac5g12791 | -1.7614 |  |  | AT1G63710.1, CYP86A7, cytochromeP450, family 86, subfamily A, polypeptide 7 |
| **14 DPI Young** |  |  |  |  |
| EL10Ac3g07108 | 2.3143 | 3 | EGF domain-specific O-linked N-acetylglucosamine transferase | AT2G41640.1, Glycosyl transferase family 61 protein |
| EL10Ac2g02928 | 1.6835 | 2 | Probable xyloglucan endotrans glucosylase/hydrolase protein 23 | AT5G57560.1, TCH4, XTH22, Xyloglucan endotransglucosylase/hydrolase family protein |
| EL10Ac8g19221 | 1.5776 | 8 | Putative beta-galactosidase | AT4G26140.1, BGAL12, beta-galactosidase 12 |
| EL10Ac9g21527 | 1.4224 |  |  | AT2G17230.1, EXL5, EXORDIUM like 5 |
| EL10Ac6g14855 | 1.4018 |  |  | AT2G23690.1 |
| EL10Ac9g22167 | 1.3487 | 9 | Protein of unknown function (DUF1005) | AT1G10020.1 |
| EL10Ac5g12589 | 1.1814 | 5 | Abscisic acid 8'-hydroxylase 1 | AT4G19230.1, CYP707A1, cytochrome P450, family 707, subfamily A, polypeptide 1 |
| EL10Ac6g13098 | 1.1730 |  |  | AT1G70090.2, GATL9, LGT8, glucosyl transferase family 8 |
| EL10Ac8g20350 | 1.1537 | 8 | Stem-specific protein TSJT1 | AT4G27450.1, Aluminium induced protein with YGL and LRDR motifs |
| EL10Ac3g06084 | 1.1477 | 3 | Probable E3 ubiquitin ligase SUD1 | AT5G38070.1, RING/FYVE/PHD zinc finger super family protein |
| EL10As5g23509 | 1.1449 |  |  | AT1G67730.1, YBR159, KCR1, ATKCR1, beta-ketoacyl reductase 1 |
| EL10Ac1g01178 | 1.1435 |  |  | AT4G08950.1, EXO, Phosphate-responsive1 family protein |
| EL10Ac4g07519 | 1.1079 | 4 | Probable LRR receptor-like serine/threonine-protein kinase At4g26540 | AT5G56040.2, Leucine-rich receptor-like protein kinase family protein |
| EL10Ac6g13662 | 1.0710 | 6 | Probable xyloglucan endotrans glucosylase/hydrolase protein 33 | AT2G01850.1, EXGT-A3, XTH27, ATXTH27, endo xyloglucan transferase A3 |
| EL10Ac9g22602 | 1.0193 | 9 | Probable glucuronoxylan glucuronosyl transferase IRX7 | AT2G28110.1, FRA8, IRX7, Exostos in family protein |
| EL10Ac8g18358 | 1.0005 | 8 | Protein of unknown function (DUF1005) | AT4G29310.1 |
| EL10Ac5g12909 | -1.0716 | 5 | Endoglucanase 6 | AT1G64390.1, AtGH9C2, GH9C2, glycosyl hydrolase 9C2 |
| EL10Ac6g13709 | -1.1277 |  |  | AT3G14735.1, U6-1, U6-1; snRNA |
| EL10Ac9g21985 | -1.1429 |  |  | AT1G80440.1, Galactose oxidase/kelch repeat super family protein |
| EL10Ac8g18366 | -1.7641 | 8 | hypothetical protein | AT4G26288.1, oxidative stress 3 |
| EL10Ac9g21200 | -1.8941 | 9 | hypothetical protein | AT5G56550.1, OXS3, ATOXS3, oxidative stress3 |
| **21 DPI Young** |  |  |  |  |
| EL10Ac5g11039 | 1.1730 | 5 | Thioredoxin-like 1-2, chloroplastic | AT5G61440.1, ACHT5, atypical CYS HIS rich thioredoxin 5 |
| **28 DPI Young** |  |  |  |  |
| EL10Ac5g10905 | 1.8915 | 5 | Probable transporter MCH1 | AT1G74780.1, Nodulin-like/Major Facilitator Super family protein |
| EL10Ac2g04827 | 1.8436 |  |  | AT3G13940.1, DNA binding; DNA-directed RNA polymerases |
| EL10Ac8g20629 | 1.7387 | 8 | Internal alternative NAD(P)H-ubiquinone oxidoreductase A2, mitochondrial | AT2G29990.1, NDA2, alternative NAD(P)H dehydrogenase 2 |
| EL10Ac3g07017 | 1.6531 | 3 | Putative disease resistance protein RGA4 | AT5G43730.1, Disease resistance protein (CC-NBS-LRR class) family |
| EL10Ac8g18366 | 1.6473 | 8 | hypothetical protein | AT4G26288.1 |
| EL10Ac5g12332 | 1.6094 | 5 | Homeobox-leucine zipper protein HAT2 | AT4G16780.1, ATHB-2, HAT4, ATHB2, HB-2, homeobox protein 2 |
| EL10Ac6g13175 | 1.5931 | 6 | Homeobox-leucine zipper protein ATHB-13 | AT1G26960.1, AtHB23, HB23, homeobox protein 23 |
| EL10Ac5g10542 | 1.5126 | 5 | hypothetical protein | AT5G11460.1, Protein of unknown function(DUF581) |
| EL10Ac3g07413 | 1.5004 |  |  | AT5G42680.2, Protein of unknown function, DUF617 |
| EL10Ac5g10693 | 1.4670 | 5 | Probable LRR receptor-like serine/threonine-protein kinase At4g26540 | AT5G48940.1, Leucine-rich repeat transmembrane protein kinase family protein |
| EL10Ac8g18493 | 1.4315 |  |  | AT5G19650.1, ATOFP8, OFP8, ovate family protein8 |
| EL10Ac8g18621 | 1.4291 | 8 | Dynein light chain, cytoplasmic | AT5G20110.1, Dynein light chain type1 family protein |
| EL10Ac3g07016 | 1.3934 | 3 | Putative disease resistance protein RGA3 | AT1G53350.1, Disease resistance protein (CC-NBS-LRR class) family |
| EL10Ac8g20617 | 1.3897 | 8 | Vacuolar protein sorting-associated protein 28 homolog 2 | AT4G21560.3, VPS28-1, vacuolar protein sorting-associated protein 28 homolog 1 |
| EL10Ac3g07459 | 1.3677 | 3 | Protein ECERIFERUM 26-like | AT4G24510.1, CER2, VC2, VC-2,v HXXXD-typeacyl-transferase family protein |
| EL10Ac4g09150 | 1.2816 | 4 | Auxin-responsive protein IAA4 | AT5G43700.1, ATAUX2 11, IAA4, AUX/IAA transcriptional regulator family protein |
| EL10Ac6g15190 | 1.2607 |  |  | AT5G65530.1, Protein kinase super family protein |
| EL10Ac2g04662 | 1.2175 | 2 | UDP-glycosyltransferase 76C2 | AT3G55710.1, UDP-Glycosyl transferase super family protein |
| EL10Ac1g00761 | 1.2089 | 1 | Serine/threonine-protein kinase D6PKL2 | AT2G44830.1, Protein kinase super family protein |
| EL10As19g24172 | 1.1797 |  |  | AT3G51050.1, FG-GAP repeat-containing protein |
| EL10Ac3g05286 | 1.1469 | 3 | Calcineurin B-like protein 4 | AT5G24270.2, SOS3, Calcium-binding EF-hand family protein |
| EL10Ac9g22303 | 1.1450 | 9 | Root cap | AT3G19430.1, late embryogenesis abundant protein-related/LEAprotein-related |
| EL10Ac4g10313 | 1.1344 | 4 | Putative lysine-specific demethylase JMJD5 | AT3G20810.1, JMJD5, 2-oxoglutarate (2OG) and Fe(II)-dependent oxygenase super family protein |
| EL10Ac6g13099 | 1.1330 |  |  | AT4G27560.1, UDP-Glycosyl transferase super family protein |
| EL10Ac9g21985 | 1.1294 |  |  | AT1G80440.1, Galactose oxidase/kelch repeat super family protein |
| EL10Ac6g13811 | 1.1154 | 6 | PB1 domain | AT3G26510.4, Octicosa peptide/Phox/Bem1p family protein |
| EL10Ac5g11039 | 1.0861 | 5 | Thioredoxin-like 1-2, chloroplastic | AT5G61440.1, ACHT5, atypical CYS HIS rich thioredoxin 5 |
| EL10Ac3g05205 | 1.0821 | 3 | Homeobox-leucine zipper protein ATHB-12 | AT3G61890.1, ATHB-12, ATHB12, HB-12, homeobox 12 |
| EL10Ac1g00725 | 1.0756 | 1 | Protein of unknown function (DUF581) | AT5G47060.1 |
| EL10Ac5g12806 | 1.0740 |  |  | AT3G61460.1, BRH1, brassinosteroid-responsive RING-H2 |
| EL10Ac8g18590 | 1.0654 | 8 | Cytochrome P450 734A1 | AT2G26710.1, BAS1, CYP734A1, CYP72B1, Cytochrome P450 super family protein |
| EL10Ac4g08054 | 1.0636 |  |  | AT1G24625.1, ZFP7, zinc finger protein 7 |
| EL10Ac5g12002 | 1.0504 | 5 | Probable xyloglucan endotransglucosylase/hydrolase protein 30 | AT1G32170.1, XTR4, XTH30, xyloglucan endotransglucosylase/hydrolase 30 |
| EL10Ac3g07015 | 1.0460 | 3 | Auxin efflux carrier component 3 | AT1G70940.1, PIN3, ATPIN3, Auxin efflux carrier family protein |
| EL10Ac3g05144 | 1.0430 |  |  | AT1G07460.1, ConcanavalinA-like lectin family protein |
| EL10Ac2g04338 | 1.0420 | 2 | Cytokinin riboside 5'-monophosphate phosphoribohydrolase LOG8 | AT5G11950.2, Putative lysine decarboxylase family protein |
| EL10Ac6g13210 | 1.0382 | 6 | Ribonuclease 1 | AT2G02990.1, RNS1, ATRNS1, ribonuclease 1 |
| EL10Ac8g18716 | 1.0318 | 8 | hypothetical protein | AT3G43955.1, transposable element gene |
| EL10Ac4g09664 | 1.0268 | 4 | Filament-like plant protein | AT1G77580.1 |
| EL10As7g23811 | 1.0249 |  |  | AT4G02100.1, Heat shock protein DnaJ with tetratricopeptide repeat |
| EL10Ac6g13908 | 1.0230 |  |  | AT5G23270.1, STP11, ATSTP11, sugar transporter 11 |
| EL10Ac5g10982 | 1.0158 | 5 | Transcription factor TCP15 | AT1G58100.1, TCP family transcription factor |
| EL10Ac8g19013 | 1.0072 | 8 | Auxin response factor 19 | AT5G20730.2, NPH4, MSG1, IAA21, ARF7, TIR5, BIP, Transcriptional factor B3 family protein/auxin-responsive factor AUX/IAA-related |
| EL10Ac5g10581 | -1.0061 | 5 | UDP-glycosyltransferase 74F2 | AT1G05680.1, UGT74E2, Uridine diphosphate glycosyltransferase 74 E2 |
| EL10Ac7g16039 | -1.0088 |  |  | AT1G07530.1, SCL14, ATGRAS2, GRAS2, SCARECROW-like14 |
| EL10Ac8g18643 | -1.0099 |  |  | AT2G47110.2, UBQ6, ubiquitin 6 |
| EL10Ac7g18174 | -1.0150 |  |  | AT4G23050.2, PAS domain-containing protein tyrosine kinase family protein |
| EL10Ac3g05105 | -1.0232 | 3 | Non-symbiotic hemoglobin 2 | AT3G10520.1, AHB2, GLB2, ARATHGLB2, NSHB2, ATGLB2, HB2, haemoglobin2 |
| EL10Ac3g05231 | -1.0271 |  |  | AT3G12590.1 |
| EL10Ac3g04978 | -1.0292 | 3 | Transcription factor bHLH25 | AT4G37850.1, basic helix-loop-helix (bHLH) DNA-binding super family protein |
| EL10Ac5g11584 | -1.0314 | 5 | Domain of unknown function (DUF3475) | AT5G24070.1, Peroxidase super family protein |
| EL10Ac8g19728 | -1.0352 | 8 | Cytochrome P450 710A1 | AT2G34490.1, CYP710A2, cytochrome P450, family 710, subfamily A, polypeptide 2 |
| EL10Ac8g18845 | -1.0454 | 8 | Alpha/beta hydrolase domain-containing protein 17B | AT2G24320.1, alpha/beta-Hydrolases super family protein |
| EL10Ac3g05382 | -1.0487 | 3 | Probable membrane-associated kinase regulator 5 | AT1G63020.2, NRPD1A, nuclear RNA polymerase D1A |
| EL10Ac7g18208 | -1.0507 | 7 | Probable disease resistance protein At1g52660 | AT5G01460.1, LMBR1-like membrane protein |
| EL10Ac4g09086 | -1.0513 | 4 | Probable WRKY transcription factor 39 | AT3G04670.1, WRKY39, ATWRKY39, WRKY DNA-binding protein39 |
| EL10Ac7g17975 | -1.0515 | 7 | Serine/threonine-protein kinase OXI1 | AT3G25250.1, AGC2-1, AGC2, OXI1, AtOXI1, AGC (cAMP dependent, cGMP-dependent and protein kinase C) kinase family protein |
| EL10Ac1g02341 | -1.0529 | 1 | Squalene monooxygenase | AT1G58440.1, XF1, SQE1, FAD/NAD(P)-binding oxidoreductase family protein |
| EL10Ac6g14811 | -1.0543 |  |  | AT5G17600.1, RING/U-box super family protein |
| EL10Ac5g12944 | -1.0546 | 5 | Auxin response factor 9 | AT3G61830.1, ARF18, auxin response factor 18 |
| EL10Ac6g14551 | -1.0573 | 6 | Proline-rich receptor-like protein kinase PERK8 | AT1G26150.1, ATPERK10, PERK10, proline-rich extensin-like receptor kinase10 |
| EL10Ac2g04042 | -1.0602 | 2 | Protein NRT1/ PTR FAMILY 1.2 | AT3G16180.1, Major facilitator superfamily protein |
| EL10Ac6g14594 | -1.0605 |  |  | AT2G15040.1, AtRLP18, RLP18, pseudogene, disease resistance protein-related, low similarity to disease resistance protein Cf-2.1 (Lycopersicon pimpinellifolium) GI:1184075; contains Pfam profile PF00560:Leucine Rich Repeat |
| EL10Ac2g02772 | -1.0622 |  |  | AT5G53220.3 |
| EL10Ac6g15451 | -1.0634 | 6 | hypothetical protein | AT3G26950.1 |
| EL10Ac9g21143 | -1.0642 | 9 | IAA-amino acid hydrolase ILR1-like 4 | AT1G51760.1, IAR3, JR3, peptidase M20/M25/M40 family protein |
| EL10Ac8g20229 | -1.0646 | 8 | Scarecrow-like protein 13 | AT5G48150.2, PAT1, GRAS family transcription factor |
| EL10Ac4g09765 | -1.0662 |  |  | AT1G64780.1, ATAMT1 ; 2, AMT1; 2, ammonium transporter 1; 2 |
| EL10Ac9g21532 | -1.0678 | 9 | Glutamate receptor 3.2 | AT4G35290.1, GLUR2, GLR3.2, ATGLR3.2, ATGLUR2, glutamate receptor 2 |
| EL10Ac3g05523 | -1.0687 |  |  | AT5G49480.1, ATCP1, CP1, Ca2+-binding protein1 |
| EL10Ac4g08909 | -1.0727 | 4 | Probable polyamine oxidase 4 | AT1G65840.1, ATPAO4, PAO4, polyamine oxidase4 |
| EL10As5g23504 | -1.0737 |  |  | AT3G05190.1, D-amino acid amino transferase-like PLP-dependent enzymes super family protein |
| EL10Ac8g18570 | -1.0738 | 8 | Serine/threonine-protein kinase Aurora-3 | AT2G45490.1, AtAUR3, AUR3, ataurora 3 |
| EL10Ac2g04844 | -1.0741 | 2 | Omega-3 fatty acid desaturase, chloroplastic | AT5G05580.1, FAD8, fatty acid desaturase 8 |
| EL10Ac9g20818 | -1.0774 | 9 | DnaJ homolog subfamily B member 13 | AT2G20560.1, DNAJ heatshock family protein |
| EL10Ac8g19863 | -1.0780 | 8 | MACPF domain-containing protein CAD1 | AT1G29690.1, CAD1, MAC/Perforin domain-containing protein |
| EL10Ac8g19626 | -1.0853 | 8 | Remorin, C-terminal region | AT1G30320.1, Remorin family protein, |
| EL10Ac8g20446 | -1.0864 | 8 | hypothetical protein | AT1G28480.1, GRX480, roxy19,Thioredoxin superfamily protein |
| EL10Ac2g02919 | -1.0884 | 2 | Probable mitochondrial chaperone BCS1-B | AT3G28540.2, P-loop containing nucleoside triphosphate hydrolases super family protein |
| EL10Ac9g22145 | -1.1005 | 9 | hypothetical protein | AT5G64510.1,TIN1 |
| EL10Ac3g05388 | -1.1066 | 3 | Anthocyanidin 5,3-O-glucosyltransferase | AT4G01070.1, GT72B1, UGT72B1, UDP Glycosyltransferase super family protein |
| EL10Ac4g09844 | -1.1092 | 4 | Probable ribose-5-phosphate isomerase 2 | AT1G71100.1, RSW10, Ribose 5-phosphate isomerase, type A protein |
| EL10Ac1g01210 | -1.1094 | 1 | Probable protein phosphatase 2C 10 | AT1G34750.1, Protein phosphatase 2C family protein |
| EL10Ac8g20094 | -1.1130 | 8 | Probable WRKY transcription factor 42 | AT4G22070.1, WRKY31, ATWRKY31, WRKY DNA-binding protein 31 |
| EL10Ac8g20010 | -1.1190 | 8 | Glucose-6-phosphate/phosphate translocator 2, chloroplastic | AT1G61800.1, GPT2, ATGPT2, glucose-6 phosphate/phosphate translocator 2 |
| EL10Ac9g22022 | -1.1316 | 9 | UDP-glycosyltransferase 82A1 | AT3G22250.1, UDP-Glycosyltransferase superfamily protein |
| EL10Ac8g18813 | -1.1330 | 8 | Tetrahydrocannabinolic acid synthase | AT4G20820.1, FAD-binding Berberine family protein |
| EL10Ac6g15549 | -1.1350 | 6 | Transcription factor RADIALIS | AT1G19510.1, ATRL5, RSM4, RL5, RAD-like5 |
| EL10Ac5g10443 | -1.1361 | 5 | Cytochrome P450 94A1 | AT2G27690.1, CYP94C1, cytochrome P450, family 94, subfamily C, polypeptide 1 |
| EL10Ac4g07616 | -1.1371 | 4 | Scarecrow-like transcription factor PAT1 | AT5G48150.2, PAT1, GRAS family transcription factor |
| EL10Ac9g21038 | -1.1388 | 9 | Aspartic proteinase-like protein 2 | AT5G22850.1, Eukaryotic aspartyl protease family protein |
| EL10Ac5g13070 | -1.1430 | 5 | Putative glucuronosyltransferase PGSIP8 | AT4G16600.1, Nucleotide-diphospho-sugar transferases super family protein |
| EL10Ac7g15884 | -1.1453 | 7 | Probable serine/threonine-protein kinase RLCKVII | AT1G07870.2, Protein kinase super family protein |
| EL10Ac2g02927 | -1.1456 | 2 | Probable xyloglucan endotransglucosylase/hydrolase protein 23 | AT5G57560.1, TCH4, XTH22, Xyloglucan endotransglucosylase/hydrolase family protein |
| EL10Ac2g04461 | -1.1475 | 2 | Receptor-like protein kinase FERONIA | AT3G51550.1, FER, Malectin/receptor-like protein kinase family protein |
| EL10Ac9g22231 | -1.1503 |  |  | AT5G42830.1, HXXXD-type acyl-transferase family protein |
| EL10Ac9g22982 | -1.1523 | 9 | Transmembrane protein 53 | AT3G19970.1, alpha/beta-Hydrolases superfamily protein |
| EL10Ac9g20986 | -1.1551 | 9 | Probable WRKY transcription factor 69 | AT3G58710.1, WRKY69, ATWRKY69, WRKY DNA-binding protein 69 |
| EL10Ac9g22161 | -1.1551 | 9 | Geraniol 8-hydroxylase | AT1G33720.1, CYP76C6, cytochrome P450, family 76, subfamily C, polypeptide 6 |
| EL10Ac5g12898 | -1.1766 | 5 | Probable WRKY transcription factor 41 | AT5G24110.1, WRKY30, ATWRKY30, WRKY DNA-binding protein 30 |
| EL10Ac5g12523 | -1.1785 | 5 | Receptor-like protein 12 | AT3G11010.1, AtRLP34, RLP34, receptor like protein 34 |
| EL10Ac3g05713 | -1.1841 | 3 | Heat stress transcription factor B-2a | AT5G62020.1, AT-HSFB2A, HSFB2A, heat shock transcription factor B2A |
| EL10Ac4g09654 | -1.1843 | 4 | Diacylglycerol kinase 2 | AT5G63770.2, ATDGK2, DGK2, diacylglycerol kinase 2 |
| EL10Ac6g15627 | -1.1911 | 6 | Calcium-transporting ATPase 2, plasma membrane-type | AT4G37640.1, ACA2, calcium ATPase 2 |
| EL10Ac5g11076 | -1.1937 | 5 | Probable LRR receptor-like serine/threonine-protein kinase At1g74360 | AT1G74360.1, Leucine-rich repeat protein kinase family protein |
| EL10Ac6g13799 | -1.1942 |  |  | AT3G26600.1, ARO4, armadillo repeat only 4 |
| EL10Ac9g21921 | -1.1956 | 9 | Glyoxalase-like domain | AT1G80160.2, Lactoyl glutathionelyase/glyoxalaseI family protein |
| EL10Ac1g00586 | -1.1965 | 1 | Germin-like protein subfamily 1 member 7 | AT3G04180.1, RmlC-like cupins super family protein |
| EL10Ac8g19962 | -1.1974 | 8 | G-type lectin S-receptor-like serine/threonine-protein kinase B120 | AT4G21390.1, B120, S-locus lectin protein kinase family protein |
| EL10As5g23601 | -1.1986 |  |  | AT1G21980.1, ATPIP5K1, ATPIPK1, PIP5K1, phosphatidylinositol-4-phosphate5-kinase 1 |
| EL10Ac4g09838 | -1.2128 | 4 | ABC transporter F family member 5 | AT5G64840.1, GCN5, ATGCN5, general control non-repressible 5 |
| EL10Ac7g16176 | -1.2176 | 7 | Phosphoinositide phospholipase C 4 | AT2G40116.1, Phosphoinositide-specific phospholipase C family protein |
| EL10Ac6g15631 | -1.2361 | 6 | BTB/POZ and TAZ domain-containing protein 4 | AT5G67480.2, BT4, BTB and TAZ domain protein 4 |
| EL10Ac9g21355 | -1.2400 | 9 | FAD-dependent urate hydroxylase | AT2G20930.1, SNARE-like super family protein |
| EL10Ac3g06960 | -1.2453 |  |  | AT4G08850.2, Leucine-rich repeat receptor-like protein kinase family protein |
| EL10Ac2g03275 | -1.2478 | 2 | Calcium-binding protein CML39 | AT5G42380.1, CML39, CML37, calmodulin like 37 |
| EL10Ac7g16846 | -1.2671 |  |  | AT2G27310.1, F-box family protein |
| EL10Ac5g12263 | -1.2681 | 5 | hAT family C-terminal dimerisation region | AT4G16950.2, RPP5, Disease resistance protein (TIR-NBS-LRR class) family |
| EL10Ac5g10401 | -1.2853 | 5 | Myb-related protein Myb4 | AT2G31180.1, ATMYB14, MYB14AT, MYB14, myb domain protein 14 |
| EL10Ac9g22880 | -1.2924 | 9 | Beta-amylase | AT4G15210.1, ATBETA-AMY, AT-BETA-AMY, RAM1, BMY1, BAM5, beta-amylase5 |
| EL10Ac9g22020 | -1.2937 | 9 | Sulfite exporter TauE/SafE | AT2G25737.1, Sulfite exporter TauE/SafE family protein |
| EL10Ac9g22325 | -1.2975 | 9 | O-acyltransferase WSD1 | AT4G08680.1, transposable element gene |
| EL10Ac1g00184 | -1.3070 | 1 | Two-component response regulator-like APRR9 | AT5G24470.1, APRR5, PRR5, pseudo-response regulator 5 |
| EL10Ac1g01565 | -1.3452 | 1 | Aspartic proteinase Asp1 | AT4G33490.2, Eukaryotic aspartyl protease family protein |
| EL10Ac8g20428 | -1.3803 | 8 | MACPF domain-containing protein NSL1 | AT1G28380.1, NSL1, MAC/Perforin domain-containing protein |
| EL10As8g23847 | -1.3822 |  |  | AT2G47330.1, P-loop containing nucleoside triphosphate hydrolases super family protein |
| EL10Ac5g12117 | -1.4000 |  |  | AT5G46760.1, Basic helix-loop-helix(bHLH) DNA-binding family protein |
| EL10Ac8g18508 | -1.4054 | 8 | Protein of unknown function (DUF1070) | AT5G48485.1, DIR1, Bifunctional inhibitor/lipid-transfer protein/seed storage2S albumin super family protein |
| EL10Ac5g12221 | -1.4058 | 5 | Probable receptor-like protein kinase At5g47070 | AT5G47070.1, Protein kinase super family protein |
| EL10Ac6g14320 | -1.4069 | 6 | Probable serine/threonine-protein kinase At1g18390 | AT1G25390.1, Protein kinase super family protein |
| EL10Ac9g21501 | -1.4093 |  |  | AT1G22370.2, AtUGT85A5, UGT85A5, UDP-glucosyl transferase 85A5 |
| EL10Ac5g10744 | -1.4104 | 5 | Probable inactive poly [ADP-ribose] polymerase SRO5 | AT1G70440.1, SRO3, similar to RC Done3 |
| EL10Ac5g12933 | -1.4307 | 5 | Uncharacterized protein At4g10930 | AT4G10940.1, RING/U-box protein |
| EL10Ac8g19291 | -1.4316 | 8 | Transcription factor bHLH25 | AT2G22770.1, NAI1, basic helix-loop-helix (bHLH) DNA-binding super family protein |
| EL10Ac8g18774 | -1.4324 | 8 | Arogenate dehydrogenase 1, chloroplastic | AT1G15710.1, prephenate dehydrogenase family protein |
| EL10Ac3g05271 | -1.4434 | 3 | Putative aminoacrylate hydrolase RutD {ECO:0000255,HAMAP-Rule:MF00832} | AT5G53050.3, alpha/beta-Hydrolases super family protein |
| EL10Ac5g11684 | -1.4506 | 5 | Transcription factor MYB44 | AT5G17800.1, AtMYB56, MYB56, mybdomain protein 56 |
| EL10Ac3g07068 | -1.4539 | 3 | Calcium-dependent protein kinase 8 | AT5G12480.1, CPK7, calmodulin-domain protein kinase 7 |
| EL10Ac9g21969 | -1.4568 | 9 | Pleiotropic drug resistance protein 1 | AT1G15520.1, PDR12, ATPDR12, ABCG40, ATABCG40, pleiotropic drugresistance12 |
| EL10Ac1g00299 | -1.4834 | 1 | Probable WRKY transcription factor 41 | AT2G46400.1, WRKY46, ATWRKY46, WRKYDNA-binding protein 46 |
| EL10Ac4g08793 | -1.4884 | 4 | hypothetical protein | AT1G43886.1, transposable element gene |
| EL10Ac7g18195 | -1.4951 | 7 | Probable disease resistance protein At1g52660 | AT5G49020.1, ATPRMT4A, PRMT4A, protein arginine methyl transferase4A |
| EL10Ac3g05314 | -1.5055 | 3 | Bark storage protein A | AT4G38120.1, ARM repeat super family protein |
| EL10Ac7g16633 | -1.5129 | 7 | Heavy-metal-associated domain | AT5G03380.1, Heavy metal transport/detoxification super family protein |
| EL10Ac7g15951 | -1.5233 | 7 | Copper transport protein ATX1 | AT5G02600.1, NAKR1, Heavy metal transport/detoxification super family protein |
| EL10Ac1g01609 | -1.5236 | 1 | Probable choline kinase 1 | AT1G71697.1, ATCK1, CK, CK1, choline kinase 1 |
| EL10Ac8g18649 | -1.5302 | 8 | 2-alkenal reductase (NADP(+)-dependent) | AT3G03080.1, Zinc-binding dehydrogenase family protein |
| EL10Ac4g09679 | -1.5395 | 4 | NAC domain-containing protein 2 | AT5G08790.1, ATAF2, anac081, NAC (No Apical Meristem) domain transcriptional regulator super family protein |
| EL10Ac7g18193 | -1.5677 | 7 | Putative disease resistance protein At4g10780 | AT1G27170.2, trans-membrane receptors; ATP binding |
| EL10Ac8g18710 | -1.5794 | 8 | TBC1 domain family member 15 | AT5G52580.1, RabGAP/TBCdomain-containing protein |
| EL10Ac9g21905 | -1.6491 | 9 | Protein LURP-one-related 12 | AT3G15810.1 |
| EL10Ac6g15140 | -1.6717 | 6 | Probable cysteine desulfurase | AT4G37100.1, Pyridoxalphosphate(PLP)-dependent transferases super family protein |
| EL10Ac3g07149 | -1.7499 | 3 | Phospho-2-dehydro-3-deoxyheptonate aldolase 1, chloroplastic | AT4G33510.2, DHS2, 3-deoxy-d-arabino-heptulosonate7-phosphate synthase |
| EL10Ac4g07807 | -1.7857 | 4 | Polygalacturonase inhibitor 1 | AT1G53430.2, Leucine-rich repeat transmembrane protein kinase |
| EL10Ac7g17931 | -1.7944 | 7 | Cytochrome P450 82G1 | AT4G37360.1, CYP81D2, cytochrome P450, family 81, subfamily D, polypeptide 2 |
| EL10Ac4g08001 | -1.8052 | 4 | Chitin-inducible gibberellin-responsive protein 1 | AT5G48150.2, PAT1, GRAS family transcription factor |
| EL10Ac4g08911 | -1.8145 | 4 | Butyrate CoA ligase AAE11, peroxisomal | AT1G65880.1, BZO1, benzoyloxyglucosinolate 1 |
| EL10Ac9g21388 | -2.0900 |  |  | AT4G38590.2, BGAL14, beta-galactosidase 14 |
| EL10Ac4g07570 | -3.1053 | 4 | Leucine-rich repeat receptor-like serine/threonine-protein kinase BAM1 | AT3G49670.1, BAM2, Leucine-rich receptor-like protein kinase family protein |
| EL10Ac9g21496 | -4.2457 |  |  | AT1G22360.1, AtUGT85A2, UGT85A2, UDP-glucosyl transferase 85 A2 |
