# Supplementary material for: Transcriptomic responses of beet to infection by beet mild yellowing virus
Source: BMC Plant Biol. 2025 Oct 21;25:1406. doi: 10.1186/s12870-025-07514-6 (PMC12538817; doi:10.1186/s12870-025-07514-6)
Supplement: Supplementary file 11 — Additional file 11. Differentially expressed genes encoding transcription factors in the resistant genotype in response to BMYV infection. [file 12870_2025_7514_MOESM11_ESM.docx]

Additional file 11. Differentially expressed genes encoding transcription factors in the resistant genotype in response to BMYV infection

| **Leaf age** | **Time point**  **(DPI)** | **Transcription factor family** | **EL10 ID** | **Arabidopsis ID** | **Log_2_foldchange** | **Arabidopsis gene description** |
| --- | --- | --- | --- | --- | --- | --- |
| Old | 21 | bHLH | EL10Ac7g16802 | At5g09750 | 1.08 | HEC3 (HECATE 3); DNA binding / transcription factor |
|  | 21 | AP2-EREBP | EL10Ac2g03544 | At2g28550 | -1.00 | RAP2.7/TOE1 (TARGET OF EAT1 1); DNA binding / transcription factor |
|  | 21 | C2C2-Dof | EL10Ac3g06110 | At3g45610 | -1.01 | Dof-type zinc finger domain-containing protein |
| Young | 28 | ARF | EL10Ac8g19013 | At5g20730 | 1.01 | NPH4 (NON-PHOTOTROPHIC HYPOCOTYL); transcription factor |
|  | 28 | C2H2 | EL10Ac4g08054 | At1g24625 | 1.06 | ZFP7 (ZINC FINGER PROTEIN 7); nucleic acid binding / transcription factor/ zinc ion binding |
|  | 28 | Homeobox | EL10Ac6g13175 | At1g26960 | 1.59 | ATHB23 (ARABIDOPSIS THALIANA HOMEOBOX PROTEIN 23) |
|  | 28 | TCP | EL10Ac5g10982 | At1g58100 | 1.01 | TCP family transcription factor, putative |
|  | 28 | ARF | EL10Ac5g12944 | At3g61830 | -1.05 | ARF18 (AUXIN RESPONSE FACTOR 18); transcription factor |
|  | 28 | bHLH | EL10Ac5g12117 | At5g46760 | -1.39 | basic helix-loop-helix (bHLH) family protein |
|  | 28 | bHLH | EL10Ac8g19291 | At2g22770 | -1.43 | NAI1; DNA binding / transcription factor |
|  | 28 | C3H | EL10Ac6g14811 | At5g17600 | -1.05 | zinc finger (C3HC4-type RING finger) family protein |
|  | 28 | GRAS | EL10Ac7g16039 | At1g07530 | -1.00 | scarecrow-like transcription factor 14 (SCL14) |
|  | 28 | GRAS | EL10Ac8g20229 | At5g48150 | -1.06 | PAT1 (PHYTOCHROME A SIGNAL TRANSDUCTION 1); transcription factor |
|  | 28 | HSF | EL10Ac3g05713 | At5g62020 | -1.18 | AT-HSFB2A (Arabidopsis thaliana heat shock transcription factor B2A) |
|  | 28 | MYB | EL10Ac5g11684 | At5g17800 | -1.45 | AtMYB56 (myb domain protein 56); DNA binding / transcription factor |
|  | 28 | MYB | EL10Ac5g10401 | At2g31180 | -1.28 | AtMYB14/Myb14at (myb domain protein 14); DNA binding / transcription factor |
|  | 28 | NAC | EL10Ac4g09679 | At5g08790 | -1.53 | ATAF2 (Arabidopsis NAC domain containing protein 81) |
