# Supplementary material for: Transcriptomic responses of beet to infection by beet mild yellowing virus
Source: BMC Plant Biol. 2025 Oct 21;25:1406. doi: 10.1186/s12870-025-07514-6 (PMC12538817; doi:10.1186/s12870-025-07514-6)
Supplement: Supplementary file 12 — Additional file 12. Biological processes, cellular components and molecular functions up- or downregulated in the resistant genotype in response to BMYV infection. [file 12870_2025_7514_MOESM12_ESM.docx]

Additional file 12. Biological processes, cellular components and molecular functions up- or downregulated in the resistant genotype in response to BMYV infection

| Time point | ID | Description | Gene count | P adj |
| --- | --- | --- | --- | --- |
| **21 DPI Old leaf** |  | **Biological processes** |  |  |
| Upregulated | GO:0042180 | cellular ketone metabolic process | 3 | 0.0127 |
|  | GO:0006979 | response to oxidative stress | 4 | 0.0127 |
|  | GO:0006986 | response to unfolded protein | 2 | 0.0127 |
|  | GO:0034620 | cellular response to unfolded protein | 2 | 0.0127 |
|  | GO:0042026 | protein refolding | 2 | 0.0127 |
|  | GO:0042181 | ketone biosynthetic process | 2 | 0.0127 |
|  | GO:0051084 | 'de novo' post-translational protein folding | 2 | 0.0127 |
|  | GO:0051085 | chaperone cofactor-dependent protein refolding | 2 | 0.0127 |
|  | GO:0006458 | 'de novo' protein folding | 2 | 0.0127 |
|  | GO:0035967 | cellular response to topologically incorrect protein | 2 | 0.0127 |
|  | GO:0009408 | response to heat | 3 | 0.0127 |
|  | GO:0035966 | response to topologically incorrect protein | 2 | 0.0131 |
|  | GO:0061077 | chaperone-mediated protein folding | 2 | 0.0172 |
|  | GO:0009699 | phenylpropanoid biosynthetic process | 2 | 0.0296 |
|  | GO:0009698 | phenylpropanoid metabolic process | 2 | 0.0467 |
| Downregulated | GO:0009642 | response to light intensity | 5 | 0.0033 |
|  | GO:0009768 | photosynthesis, light harvesting in photosystem I | 2 | 0.0190 |
|  | GO:0009765 | photosynthesis, light harvesting | 2 | 0.0486 |
|  |  | **Cellular components** |  |  |
| Downregulated | GO:0009534 | chloroplast thylakoid | 5 | 0.0018 |
|  | GO:0031976 | plastid thylakoid | 5 | 0.0018 |
|  | GO:0009579 | thylakoid | 5 | 0.0028 |
|  | GO:0009523 | photosystem II | 2 | 0.0073 |
|  | GO:0009521 | photosystem | 2 | 0.0116 |
|  | GO:0009535 | chloroplast thylakoid membrane | 3 | 0.0259 |
|  | GO:0055035 | plastid thylakoid membrane | 3 | 0.0259 |
|  | GO:0042651 | thylakoid membrane | 3 | 0.0259 |
|  | GO:0034357 | photosynthetic membrane | 3 | 0.0259 |
|  | GO:0042170 | plastid membrane | 3 | 0.0416 |
|  |  | **Molecular function** |  |  |
| Upregulated | GO:0051787 | misfolded protein binding | 2 | 0.0051 |
|  | GO:0044183 | protein folding chaperone | 2 | 0.0051 |
|  | GO:0031072 | heat shock protein binding | 2 | 0.0088 |
|  | GO:0051082 | unfolded protein binding | 2 | 0.0357 |
| Downregulated |  |  |  |  |
|  | GO:0016168 | chlorophyll binding | 2 | 0.0126 |
| **14 DPI Young leaf** |  | **Biological processes** |  |  |
| Upregulated | GO:0010410 | hemicellulose metabolic process | 3 | 0.0031 |
|  | GO:0042546 | cell wall biogenesis | 4 | 0.0039 |
|  | GO:0010383 | cell wall polysaccharide metabolic process | 3 | 0.0039 |
|  | GO:0044036 | cell wall macromolecule metabolic process | 3 | 0.0051 |
|  | GO:0010411 | xyloglucan metabolic process | 2 | 0.0067 |
|  | GO:0071695 | anatomical structure maturation | 3 | 0.0122 |
|  | GO:0021700 | developmental maturation | 3 | 0.0130 |
|  | GO:0044264 | cellular polysaccharide metabolic process | 3 | 0.0130 |
|  | GO:0005976 | polysaccharide metabolic process | 3 | 0.0279 |
|  | GO:0044262 | cellular carbohydrate metabolic process | 3 | 0.0279 |
|  | GO:0071669 | plant-type cell wall organization or biogenesis | 3 | 0.0301 |
|  | GO:0010015 | root morphogenesis | 3 | 0.0352 |
|  | GO:0036294 | cellular response to decreased oxygen levels | 2 | 0.0368 |
|  | GO:0071453 | cellular response to oxygen levels | 2 | 0.0368 |
|  | GO:0071456 | cellular response to hypoxia | 2 | 0.0368 |
| Downregulated | GO:0046688 | response to copper ion | 1 | 0.0431 |
|  | GO:2000070 | regulation of response to water deprivation | 1 | 0.0431 |
|  | GO:0009956 | radial pattern formation | 1 | 0.0431 |
|  | GO:0010043 | response to zinc ion | 1 | 0.0431 |
|  | GO:0010218 | response to far red light | 1 | 0.0431 |
|  | GO:2000028 | regulation of photoperiodism, flowering | 1 | 0.0485 |
|  | GO:0010114 | response to red light | 1 | 0.0485 |
|  | GO:0046686 | response to cadmium ion | 1 | 0.0485 |
|  | GO:0009813 | flavonoid biosynthetic process | 1 | 0.0485 |
|  |  | **Cellular components** |  |  |
| Upregulated | GO:0009505 | plant-type cell wall | 2 | 0.0376 |
|  | GO:0005618 | cell wall | 2 | 0.0376 |
|  | GO:0030312 | external encapsulating structure | 2 | 0.0376 |
| Downregulated | GO:0016607 | nuclear speck | 1 | 0.0276 |
|  | GO:0016604 | nuclear body | 1 | 0.0276 |
|  |  | **Molecular function** |  |  |
| Upregulated | GO:0016762 | xyloglucan:xyloglucosyl transferase activity | 2 | 0.0022 |
|  | GO:0016757 | glycosyltransferase activity | 4 | 0.0029 |
|  | GO:0004553 | hydrolase activity, hydrolyzing O-glycosyl compounds | 3 | 0.0054 |
|  | GO:0016758 | hexosyltransferase activity | 3 | 0.0054 |
|  | GO:0030247 | polysaccharide binding | 2 | 0.0054 |
|  | GO:0016798 | hydrolase activity, acting on glycosyl bonds | 3 | 0.0054 |
|  | GO:0046527 | glucosyltransferase activity | 2 | 0.0159 |
|  | GO:0030246 | carbohydrate binding | 2 | 0.0170 |
|  | GO:0001653 | peptide receptor activity | 1 | 0.0345 |
|  | GO:0004565 | beta-galactosidase activity | 1 | 0.0345 |
|  | GO:0015020 | glucuronosyltransferase activity | 1 | 0.0377 |
|  | GO:0015925 | galactosidase activity | 1 | 0.0377 |
| Downregulated | GO:0008810 | cellulase activity | 1 | 0.0118 |
| **21 DPI Young leaf** |  | **Biological processes** |  |  |
| Upregulated | GO:0045454 | cell redox homeostasis | 1 | 0.0067 |
|  | GO:0019725 | cellular homeostasis | 1 | 0.0189 |
|  | GO:0042592 | homeostatic process | 1 | 0.0286 |
| **28 DPI Young leaf** |  | **Biological processes** |  |  |
| Upregulated | GO:0010015 | root morphogenesis | 5 | 0.0365 |
|  | GO:0009638 | phototropism | 2 | 0.0365 |
|  | GO:0009606 | tropism | 3 | 0.0365 |
|  | GO:0009733 | response to auxin | 4 | 0.0365 |
|  | GO:0010082 | regulation of root meristem growth | 2 | 0.0365 |
| Downregulated | GO:0014070 | response to organic cyclic compound | 16 | 1.16E-06 |
|  | GO:0009751 | response to salicylic acid | 12 | 1.16E-06 |
|  | GO:0009753 | response to jasmonic acid | 10 | 0.0015 |
|  | GO:0070542 | response to fatty acid | 10 | 0.0015 |
|  | GO:0071407 | cellular response to organic cyclic compound | 7 | 0.0062 |
|  | GO:0071446 | cellular response to salicylic acid stimulus | 5 | 0.0062 |
|  | GO:0006979 | response to oxidative stress | 9 | 0.0112 |
|  | GO:0010193 | response to ozone | 3 | 0.0128 |
|  | GO:0009863 | salicylic acid mediated signaling pathway | 4 | 0.0176 |
|  | GO:0009867 | jasmonic acid mediated signaling pathway | 5 | 0.0176 |
|  | GO:0071395 | cellular response to jasmonic acid stimulus | 5 | 0.0176 |
|  | GO:0071398 | cellular response to fatty acid | 5 | 0.0176 |
|  | GO:0036294 | cellular response to decreased oxygen levels | 5 | 0.0176 |
|  | GO:0071453 | cellular response to oxygen levels | 5 | 0.0176 |
|  | GO:0071456 | cellular response to hypoxia | 5 | 0.0176 |
|  | GO:0009611 | response to wounding | 9 | 0.0199 |
|  |  | **Molecular function** |  |  |
| Upregulated | GO:0019900 | kinase binding | 3 | 0.0222 |
| Downregulated | GO:0004674 | protein serine/threonine kinase activity | 3 | 0.0390 |
