# Supplementary material for: Transcriptomic responses of beet to infection by beet mild yellowing virus
Source: BMC Plant Biol. 2025 Oct 21;25:1406. doi: 10.1186/s12870-025-07514-6 (PMC12538817; doi:10.1186/s12870-025-07514-6)
Supplement: Supplementary file 13 — Additional file 13. Biological processes, cellular components and molecular functions up- or downregulated in the susceptible genotype in response to BMYV infection. [file 12870_2025_7514_MOESM13_ESM.docx]

Additional file 13. Biological processes, cellular components and molecular functions up- or downregulated at different in the susceptible genotype in response to BMYV infection

| **Time point** | **ID** | | **Description** | **Gene count** | **P adjust** |
| --- | --- | --- | --- | --- | --- |
| **14 DPI Old leaf** |  | | **Biological processes** |  |  |
| **Upregulated** | GO:0006073 | | cellular glucan metabolic process | 11 | 0.0017 |
|  | GO:0044042 | | glucan metabolic process | 11 | 0.0017 |
|  | GO:0044264 | | cellular polysaccharide metabolic process | 13 | 0.0017 |
|  | GO:0044262 | | cellular carbohydrate metabolic process | 15 | 0.0028 |
|  | GO:0044036 | | cell wall macromolecule metabolic process | 9 | 0.0066 |
|  | GO:0005976 | | polysaccharide metabolic process | 14 | 0.0075 |
|  | GO:0010410 | | hemicellulose metabolic process | 6 | 0.0257 |
|  | GO:0019748 | | secondary metabolic process | 15 | 0.0335 |
|  | GO:0010411 | | xyloglucan metabolic process | 4 | 0.0335 |
|  | GO:0010383 | | cell wall polysaccharide metabolic process | 7 | 0.0335 |
| **Downregulated** | GO:0009768 | | photosynthesis, light harvesting in photosystem I | 8 | 7.60E-09 |
|  | GO:0009765 | | photosynthesis, light harvesting | 9 | 6.14E-08 |
|  | GO:0019684 | | photosynthesis, light reaction | 14 | 1.73E-07 |
|  | GO:0015979 | | photosynthesis | 16 | 4.55E-07 |
|  | GO:0006091 | | generation of precursor metabolites and energy | 14 | 0.0035 |
|  | GO:0009644 | | response to high light intensity | 6 | 0.0035 |
|  | GO:0009409 | | response to cold | 13 | 0.0103 |
|  | GO:0046688 | | response to copper ion | 3 | 0.01802 |
|  | GO:0009642 | | response to light intensity | 11 | 0.0233 |
|  | GO:0010037 | | response to carbon dioxide | 3 | 0.0259 |
|  | GO:0009767 | | photosynthetic electron transport chain | 5 | 0.0259 |
|  | GO:0015994 | | chlorophyll metabolic process | 7 | 0.0286 |
|  | GO:0010218 | | response to far red light | 4 | 0.0332 |
|  | GO:0010196 | | nonphotochemical quenching | 3 | 0.0348 |
|  | GO:1990066 | | energy quenching | 3 | 0.0348 |
|  | GO:0010118 | | stomatal movement | 7 | 0.0375 |
|  | GO:0033013 | | tetrapyrrole metabolic process | 9 | 0.0409 |
|  | GO:0010114 | | response to red light | 5 | 0.0420 |
|  |  | | **Cellular components** |  |  |
| **Upregulated** | GO:0009505 | | plant-type cell wall | 14 | 0.0023 |
|  | GO:0005618 | | cell wall | 14 | 0.0023 |
|  | GO:0030312 | | external encapsulating structure | 14 | 0.0023 |
|  | GO:0031226 | | intrinsic component of plasma membrane | 8 | 0.0206 |
| **Downregulated** | GO:0009579 | | thylakoid | 26 | 2.01E-09 |
|  | GO:0034357 | | photosynthetic membrane | 22 | 2.01E-09 |
|  | GO:0055035 | | plastid thylakoid membrane | 21 | 2.01E-09 |
|  | GO:0042651 | | thylakoid membrane | 21 | 5.80E-09 |
|  | GO:0009535 | | chloroplast thylakoid membrane | 20 | 5.80E-09 |
|  | GO:0042170 | | plastid membrane | 23 | 8.93E-09 |
|  | GO:0009534 | | chloroplast thylakoid | 22 | 1.55E-08 |
|  | GO:0031976 | | plastid thylakoid | 22 | 1.65E-08 |
|  | GO:0098807 | | chloroplast thylakoid membrane protein complex | 6 | 1.93E-06 |
|  | GO:0009521 | | photosystem | 6 | 0.0004 |
|  | GO:0009941 | | chloroplast envelope | 16 | 0.0027 |
|  | GO:0009523 | | photosystem II | 4 | 0.0072 |
|  | GO:0009522 | | photosystem I | 3 | 0.0072 |
|  |  | | **Molecular function** |  |  |
| **Upregulated** | GO:0046527 | | glucosyltransferase activity | 10 | 0.0007 |
|  | GO:0016798 | | hydrolase activity, acting on glycosyl bonds | 13 | 0.0025 |
|  | GO:0004553 | | hydrolase activity, hydrolyzing O-glycosyl compounds | 12 | 0.0025 |
|  | GO:0016758 | | hexosyltransferase activity | 12 | 0.0025 |
|  | GO:0016762 | | xyloglucan:xyloglucosyl transferase activity | 4 | 0.0025 |
|  | GO:0030247 | | polysaccharide binding | 6 | 0.0043 |
|  | GO:0016620 | | oxidoreductase activity | 4 | 0.0209 |
|  | GO:0015112 | | nitrate transmembrane transporter activity | 3 | 0.0304 |
|  | GO:0030246 | | carbohydrate binding | 7 | 0.0390 |
|  | GO:0020037 | | heme binding | 9 | 0.0390 |
|  | GO:0016903 | | oxidoreductase activity, acting on the aldehyde or oxo group of donors | 4 | 0.0464 |
| **Downregulated** | GO:0016168 | | chlorophyll binding | 7 | 4.15E-07 |
|  | GO:0016717 | | oxidoreductase activity | 3 | 0.0444 |
| **21 DPI Old leaf** |  | | **Biological processes** |  |  |
| **Upregulated** | GO:0006259 | | DNA metabolic process | 33 | 1.48E-14 |
|  | GO:0006260 | | DNA replication | 20 | 1.48E-14 |
|  | GO:0006261 | | DNA-templated DNA replication | 18 | 2.35E-13 |
|  | GO:0006281 | | DNA repair | 25 | 3.14E-13 |
|  | GO:0006974 | | cellular response to DNA damage stimulus | 26 | 3.75E-13 |
|  | GO:0000724 | | double-strand break repair via homologous recombination | 14 | 1.00E-10 |
|  | GO:0000725 | | recombinational repair | 14 | 1.98E-10 |
|  | GO:0006310 | | DNA recombination | 17 | 1.98E-10 |
|  | GO:0006268 | | DNA unwinding involved in DNA replication | 8 | 3.66E-10 |
|  | GO:0032392 | | DNA geometric change | 9 | 7.15E-10 |
|  | GO:0032508 | | DNA duplex unwinding | 9 | 7.15E-10 |
|  | GO:0006302 | | double-strand break repair | 15 | 7.15E-10 |
|  | GO:0071103 | | DNA conformation change | 9 | 9.88E-09 |
|  | GO:0006270 | | DNA replication initiation | 7 | 5.05E-07 |
|  | GO:0051276 | | chromosome organization | 19 | 1.27E-06 |
|  | GO:0022402 | | cell cycle process | 22 | 1.91E-06 |
|  | GO:0000727 | | double-strand break repair via break-induced replication | 5 | 7.88E-06 |
|  | GO:0000278 | | mitotic cell cycle | 13 | 4.98E-05 |
|  | GO:1903047 | | mitotic cell cycle process | 11 | 0.0002 |
|  | GO:1902969 | | mitotic DNA replication | 4 | 0.0002 |
|  | GO:0033260 | | nuclear DNA replication | 4 | 0.0005 |
|  | GO:0000076 | | DNA replication checkpoint signaling | 4 | 0.0006 |
|  | GO:0006271 | | DNA strand elongation involved in DNA replication | 4 | 0.0008 |
|  | GO:0022616 | | DNA strand elongation | 4 | 0.0008 |
|  | GO:0000819 | | sister chromatid segregation | 6 | 0.0025 |
|  | GO:0098813 | | nuclear chromosome segregation | 7 | 0.0037 |
|  | GO:0031570 | | DNA integrity checkpoint signaling | 4 | 0.0043 |
|  | GO:0044786 | | cell cycle DNA replication | 5 | 0.0048 |
|  | GO:0007059 | | chromosome segregation | 7 | 0.0053 |
|  | GO:0010948 | | negative regulation of cell cycle process | 5 | 0.0068 |
|  | GO:1902750 | | negative regulation of cell cycle G2/M phase transition | 3 | 0.0087 |
|  | GO:0045005 | | DNA-templated DNA replication maintenance of fidelity | 3 | 0.0108 |
|  | GO:0045786 | | negative regulation of cell cycle | 5 | 0.0108 |
|  | GO:0007062 | | sister chromatid cohesion | 4 | 0.0142 |
|  | GO:0000075 | | cell cycle checkpoint signaling | 4 | 0.0194 |
|  | GO:0006289 | | nucleotide-excision repair | 4 | 0.0203 |
|  | GO:1901988 | | negative regulation of cell cycle phase transition | 4 | 0.0203 |
|  | GO:0010564 | | regulation of cell cycle process | 6 | 0.0248 |
|  | GO:0006284 | | base-excision repair | 3 | 0.0278 |
|  | GO:0006298 | | mismatch repair | 3 | 0.0278 |
|  | GO:1902749 | | regulation of cell cycle G2/M phase transition | 3 | 0.0478 |
| **Upregulated** |  | | **Cellular components** |  |  |
|  | GO:0005694 | | chromosome | 22 | 5.37E-11 |
|  | GO:0043596 | | nuclear replication fork | 8 | 1.54E-09 |
|  | GO:0005657 | | replication fork | 8 | 1.23E-07 |
|  | GO:0030894 | | replisome | 6 | 1.23E-07 |
|  | GO:0043601 | | nuclear replisome | 6 | 1.23E-07 |
|  | GO:0032993 | | protein-DNA complex | 7 | 5.58E-06 |
|  | GO:0042575 | | DNA polymerase complex | 5 | 1.55E-05 |
|  | GO:0000228 | | nuclear chromosome | 9 | 4.42E-05 |
|  | GO:0000347 | | THO complex | 4 | 0.0001 |
|  | GO:1990391 | | DNA repair complex | 3 | 0.0040 |
|  | GO:0098687 | | chromosomal region | 4 | 0.0062 |
|  | GO:0000785 | | chromatin | 7 | 0.0069 |
|  | GO:0000775 | | chromosome, centromeric region | 3 | 0.0310 |
|  | GO:0035861 | | site of double-strand break | 2 | 0.0375 |
|  | GO:0090734 | | site of DNA damage | 2 | 0.0375 |
| **Upregulated** |  | | **Molecular function** |  |  |
|  | GO:0140097 | | catalytic activity, acting on DNA | 15 | 2.09521E-07 |
|  | GO:0043138 | | 3'-5' DNA helicase activity | 6 | 8.53251E-07 |
|  | GO:0003678 | | DNA helicase activity | 8 | 2.69716E-06 |
|  | GO:0003697 | | single-stranded DNA binding | 8 | 6.36372E-05 |
|  | GO:0008094 | | ATP-dependent activity, acting on DNA | 8 | 8.9701E-05 |
|  | GO:0017116 | | single-stranded DNA helicase activity | 4 | 0.000153673 |
|  | GO:0140640 | | catalytic activity, acting on a nucleic acid | 19 | 0.000181178 |
|  | GO:0004386 | | helicase activity | 9 | 0.0003 |
|  | GO:0003682 | | chromatin binding | 9 | 0.0015 |
|  | GO:0020037 | | heme binding | 10 | 0.0051 |
|  | GO:0140657 | | ATP-dependent activity | 16 | 0.0051 |
|  | GO:0003688 | | DNA replication origin binding | 3 | 0.0051 |
|  | GO:0046906 | | tetrapyrrole binding | 10 | 0.0092 |
|  | GO:0030983 | | mismatched DNA binding | 3 | 0.0094 |
|  | GO:0003887 | | DNA-directed DNA polymerase activity | 3 | 0.0303 |
|  | GO:0034061 | | DNA polymerase activity | 3 | 0.0421 |
| **14 DPI Young leaf** | |  | **Biological processes** |  |  |
| **Downregulated** | GO:0010218 | | response to far red light | 3 | 0.0014 |
|  | GO:0009639 | | response to red or far red light | 5 | 0.0014 |
|  | GO:0045892 | | negative regulation of transcription, DNA-templated | 3 | 0.0323 |
|  | GO:1902679 | | negative regulation of RNA biosynthetic process | 3 | 0.0323 |
|  | GO:1903507 | | negative regulation of nucleic acid-templated transcription | 3 | 0.0323 |
|  | GO:0051253 | | negative regulation of RNA metabolic process | 3 | 0.0343 |
|  | GO:0045934 | | negative regulation of nucleobase-containing compound metabolic process | 3 | 0.0464 |
|  | GO:0010558 | | negative regulation of macromolecule biosynthetic process | 3 | 0.0471 |
| **28 DPI Young leaf** | | | **Biological processes** |  |  |
| **Downregulated** | GO:0010114 | | response to red light | 2 | 0.0471 |
|  | GO:0009639 | | response to red or far red light | 5 | 0.0039 |
|  | GO:0071483 | | cellular response to blue light | 2 | 0.0398 |
